# *Drosophila* Neurotrophins Reveal a Common Mechanism for Nervous System Formation

**DOI:** 10.1371/journal.pbio.0060284

**Published:** 2008-11-18

**Authors:** Bangfu Zhu, Jenny A Pennack, Peter McQuilton, Manuel G Forero, Kenji Mizuguchi, Ben Sutcliffe, Chun-Jing Gu, Janine C Fenton, Alicia Hidalgo

**Affiliations:** 1 Neurodevelopment Group, School of Biosciences, University of Birmingham, Birmingham, United Kingdom; 2 Department of Genetics, University of Cambridge, Cambridge, United Kingdom; 3 Department of Biochemistry, University of Cambridge, Cambridge, United Kingdom; 4 Department of Applied Mathematics and Theoretical Physics, Centre for Mathematical Sciences, University of Cambridge, United Kingdom; 5 National Institute of Biomedical Innovation, Osaka, Japan; University of Cambridge, United Kingdom

## Abstract

Neurotrophic interactions occur in *Drosophila*, but to date, no neurotrophic factor had been found. Neurotrophins are the main vertebrate secreted signalling molecules that link nervous system structure and function: they regulate neuronal survival, targeting, synaptic plasticity, memory and cognition. We have identified a neurotrophic factor in flies, *Drosophila* Neurotrophin (DNT1), structurally related to all known neurotrophins and highly conserved in insects. By investigating with genetics the consequences of removing DNT1 or adding it in excess, we show that DNT1 maintains neuronal survival, as more neurons die in *DNT1* mutants and expression of *DNT1* rescues naturally occurring cell death, and it enables targeting by motor neurons. We show that Spätzle and a further fly neurotrophin superfamily member, DNT2, also have neurotrophic functions in flies. Our findings imply that most likely a neurotrophin was present in the common ancestor of all bilateral organisms, giving rise to invertebrate and vertebrate neurotrophins through gene or whole-genome duplications. This work provides a missing link between aspects of neuronal function in flies and vertebrates, and it opens the opportunity to use *Drosophila* to investigate further aspects of neurotrophin function and to model related diseases.

## Author Summary

Neurotrophins are secreted proteins that link nervous system structure and function in vertebrates. They regulate neuronal survival, thus adjusting cell populations, and connectivity, enabling the formation of neuronal circuits. They also regulate patterns of dendrites and axons, synaptic function, memory, learning, and cognition; and abnormal neurotrophin function underlies psychiatric disorders. Despite such relevance for nervous system structure and function, neurotrophins have been missing from invertebrates. We show here the identification and functional demonstration of a neurotrophin family in the fruit fly, *Drosophila*. Our findings imply that the neurotrophins may be present in all animals with a centralised nervous system (motor and sensory systems) or brain, supporting the notion of a common origin for the brain in evolution. This work bridges a void in the understanding of the *Drosophila* and human nervous systems, and it opens the opportunity to use the powerful fruit fly for neurotrophin related studies.

## Abstract 3

Members of the neurotrophin superfamily mediate critical roles in neuronal survival and targeting in the fruit fly*Drosophila*. Although this is an accepted role for neurotrophins in vertebrates, scant previous evidence has been able to demonstrate such a conserved role in invertebrates.

## Introduction

In vertebrate brain development, neurons are produced in excess, and surplus neurons are eliminated through apoptosis (cell death), adjusting innervation, targeting, and connectivity to target size [1]. Neurotrophins (NTs) are the major class of molecules promoting neuronal survival in vertebrates. They also control cell proliferation and neuronal differentiation, and they are required for axonal and dendritic elaborations, synaptic plasticity, excitability, and long-term potentiation (LTP, the basis of memory and learning) [2–5]. NTs underlie most aspects of vertebrate nervous system development and function, and abnormal NT function is linked to psychiatric disorders [6–9]. NTs are the key molecules linking nervous system structure and function in vertebrates [2,3]. Despite such fundamental roles, NTs have been missing from invertebrates.

There is compelling evidence that neurotrophic factors exist in *Drosophila*. As in vertebrates, about half the neurons die in the fruit fly central nervous system (CNS) during embryogenesis [10]. Apoptosis occurs in most neuroblast lineages [11,12], and there is dramatic hyperplasia in mutant embryos lacking programmed cell death [13]. In multiple CNS contexts, the survival of subsets of neurons and glia requires long-range, nonautonomous support. For instance, there are no glial cells of retinal origin; glia enter the retina through the optic stalk, and if they are defective, such as in *repo* mutants, retinal neurons die in excess [14]. In *disconnected* mutants, the optic lobes (where the retinal photoreceptor neurons project to in the brain) degenerate. When mosaic clones of *disconnected* mutant cells are generated in the brain optic lobes in otherwise normal flies, retinal neurons die [15]. Lack of connectivity at the optic lobe also results in massive optic lobe neuronal death [16,17] due to abnormal function originating from the retina rather than the brain [16,17]. A trophic factor for retinal neurons is predicted to emanate from the brain optic lobe glia [16,17]. In the embryonic CNS, upon glial ablation or mutations in *glial cells missing*, there is excess neuronal apoptosis [18]. Glia are also produced in excess: most dramatically, in the embryo, 75% midline glia and a small subset of longitudinal glia die during axon guidance (prior to homeostatic functions of glia) [19–24]. Identified gliatrophic factors include the neuregulin homolog Vein [24] and the TGFα homolog Spitz [19,25,26], both ligands of EGFR, and the ligands of the PDGR homolog PVR [27]. Other properties commonly assigned to complex brains and to NT function, such as synaptic plasticity, LTP, and complex behaviour, all occur in flies. However, no neurotrophic factor has been identified in *Drosophila*.

The NTs comprise brain-derived neurotrophic factor (BDNF), nerve growth factor (NGF), NT3, and NT4/5 (plus NT6/7 in fish) and bind the Receptor Tyrosine Kinases TrkA, -B, -C, the atypical TNFR superfamily member p75, and Integrin α9β1 [28–30]. Pro-NTs bind p75 to promote cell death, and mature NTs bind Trk and p75 receptors, or p75 alone, to promote cell survival [3,28,30]. Vertebrate NTs bind Trks to activate the MAPKinase/ERK and AKT pathways (promoting cell survival), PLC-γ (regulating calcium levels), and NFκB (promoting cell survival) [3,30]. Binding of NTs to p75 independently of Trks results in cell death or cell survival, through JNK and NFκB, respectively [30]. In an evolutionary context, p75 is more ancient than the Trks [30]. The most conserved NT among vertebrates is BDNF, and *BDNF* mutations correlate with epilepsy, anxiety, depression, attention deficit disorder, autism, and other cognitive and psychiatric disorders (e.g., [6–9]). NTs underlie an endogenous mechanism of CNS repair [31], and disregulation of NGF underlies chronic pain (e.g., in cancer) [32]. *Drosophila* is a very powerful model organism used to understand gene networks and model disease; however, a surprising void has been the lack of NT studies in flies.

NT ligands and receptors have been identified throughout the invertebrate deuterostomes (Figure S1). There are functional Trk receptors in the lancelet *Amphioxus* [33], and *p75* and *Trk* orthologs have been identified in sea urchin and acorn worm [34–36]. Searches of sequenced genomes have revealed NTs in all deuterostome groups, represented by *Amphioxus* NT (*Bf-NT*), acorn worm NT (*Sk-NT*), and sea urchin NT (*Sp-NT*) [34,37,38] (Figure S1 and Table S1). In protostome invertebrates, a bona fide Trk (in the snail *Lymnea*) and an atypical Trk (in the snail *Aplysia*), have also been identified in molluscs [35,36,39]. The function of these ancient NTs and receptors is unknown. These findings indicate that NTs are more ancient in evolution than previously thought, although no NT has been identified in protostomes.

The presence of NTs in flies has been controversial. Structural and biochemical features of the *Drosophila* protein Spätzle (Spz) revealed an NGF domain [40,41]. However, a parallel similarity to horseshoe crab coagulogen, involved in the blood-clotting cascade [41], overshadowed that earlier finding. An initial computational analysis of the sequenced genomes based on BLAST searches declared lack of NTs in flies [42]. However, this simple BLAST search missed 30% of *Drosophila* genes and would have missed any proteins with structural conservation despite sequence divergence. Structural predictions have confirmed that Spz belongs to the NT superfamily [43]. There are to date no functional studies of Spz in the CNS, so whether it plays neurotrophic roles is unknown.

To investigate whether a NT may underlie some of the structural and functional aspects of the insect nervous system, we searched the sequenced *Drosophila* genome for NT sequences. We show here that *Drosophila* Neurotrophin 1 (DNT1) is a NT superfamily member that promotes neuronal survival and targeting, and that there is a NT family in *Drosophila* formed by DNT1, DNT2, and Spz.

## Results

### Identification of DNT1

**Figure 1:**
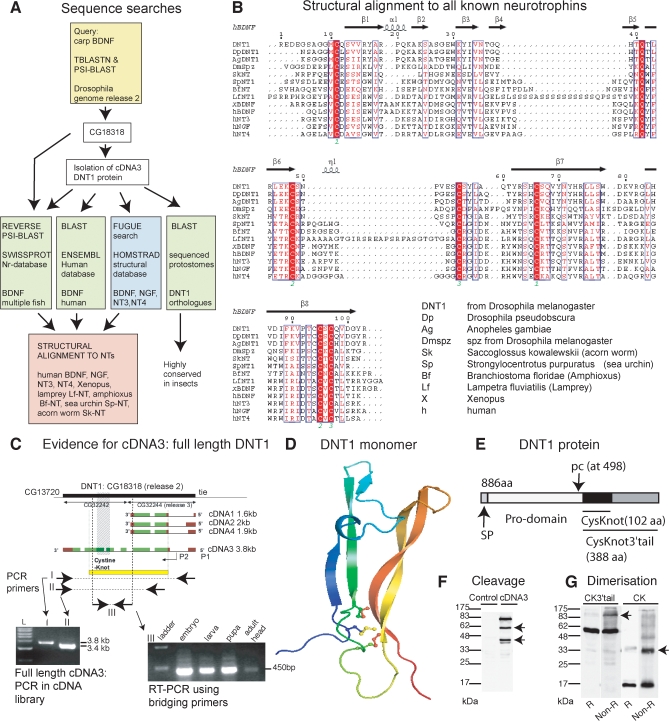
Identification of a *Drosophila* Neurotrophin (A) Bioinformatic searches used to identify DNT1. (B) Sequence-structure alignment of the Cysknot domains of DNT1 and representative neurotrophins: Arrows: β-strands; spirals: α-helices; identical residues are shown in white over red (6 Cys and Gln conserved in all NTs); conservative substitutions in the hydrophic core in red. (C) Evidence for the existence of cDNA3 spanning the pro- and Cysknot domains. DNT1 locus showing exons (green), introns (gaps), and UTRs (red), Cysknot domain (shaded). P1, P2: predicted promoters. Yellow box indicates the region deleted in the *DNT1^41^* and *DNT1^55^* mutant alleles. cDNA3 was amplified by PCR on a larval/pupal cDNA library using primers to the 5′ untranslated end of *CG18318* or from the ATG and to the 3′ untranslated end of *CG18318*. RT-PCR was carried out on RNA using bridging primers: 5′ primers were located at the 3′ of cDNA1 from *CG32244*, and 3′ primers were located at the 3′ end of the Cysknot domain. Amplification by RT-PCR with these primers is only possible if an mRNA spanning both *CG32244* and *CG32242* exists. (D) Model of the DNT1 protomer: blue is N-terminus, red is C-terminus, predicted disulphide bonds shown as ball-and-stick model. (E) DNT1 protein. SP: signal peptide; p.c: predicted cleavage site at position 498; Cysknot (black). (F) Cleavage products (arrows) of DNT1(cDNA3-V5) detected with anti-V5 in S2 cells. (G) The DNT1 Cysknot (CK) or Cysknot3′tail (CK3′tail) form dimers (arrows) when expressed in S2 cells: western blots showing tagged SP-CK-V5 and SP-CK3′tail-V5 detected with anti-V5 antibodies run under reducing (R) and nonreducing (Non-R) conditions. The dimers dissociate into monomers in reducing conditions.

We used 28 known full-length and Cystine-knot domain (Cysknot, characteristic of NTs) vertebrate NT sequences to query release 2 of the *Drosophila* genome with TBLASTN and PSI-BLAST, which is specific to detect distantly related sequences (Figure 1A and Text S1). When using carp BDNF as query, both searches identified *CG18318*. In turn, CG18318 identified BDNF from multiple species, from fish to human. After isolating the full-length cDNA3 from *CG18318* (Figures 1C and S2; GenBank accession number: FJ172423), we used the protein sequence to carry out a structure-based search using FUGUE (Figure 1A) [44]. FUGUE identifies distantly related proteins, the sequence of which may have diverged through evolution while retaining structural conservation [44]. FUGUE compares the query protein sequence with the HOMSTRAD database of proteins of known structure, and it assigns amino-acid substitutions a score depending on how this affects protein structure [44]. FUGUE identified the human NTs with over 99% certainty as probable homologs of cDNA3 from *CG18318* (Table S2), above similarity to coagulogen. Search of the ENSEMBL human database using cDNA3 protein sequence as query also identified human BDNF (Figure 1A). Thus, we named the protein encoded by cDNA3 *Drosophila* Neurotrophin1 (DNT1). PSI-BLAST searches using Spz as query to the *Drosophila* genome had identified distant *spz* paralogs [45]: *DNT1* is *spätzle 2* (*spz2*).

**Figure 2:**
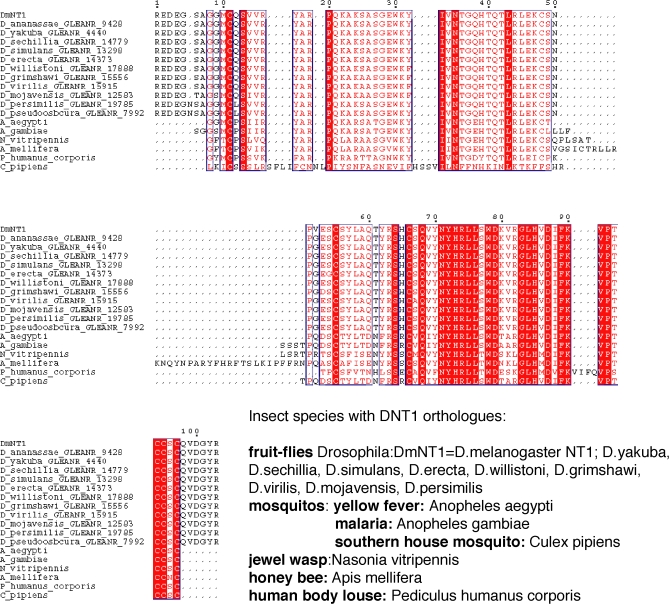
*DNT1* Is Highly Conserved in Insects Alignment of the DNT1 Cysknot domain plus some flanking sequences to orthologs from insects with sequenced genomes, including 12 *Drosophila* species, three mosquito species (*Anopheles* and *Culex*), honey bee (*Apis*), jewel wasp (*Nasonia*), and human body louse (*Pediculus*). Identical residues are shown in white over red; conservative substitutions in red. DNT1 is very highly conserved throughout the whole protein sequence in all *Drosophila* species (full sequence not shown, but note sequences flanking the Cysknot) and very highly conserved within the Cysknot in all insects, but outside *Drosophila*, the sequences outside the Cysknot diverge.

To verify the structural features of DNT1, we carried out a structural alignment of DNT1 to known NT sequences from human, *Xenopus*, and the ancient NTs from lamprey (Lf-NT), *Amphioxus* (Bf-NT), sea urchin (Sp-NT), and acorn worm (Sk-NT) (Figure 1B). All the essential residues that form the NT Cysknot (positions 499–601 in DNT1) are conserved in all these sequences, i.e., the six cysteines, the glutamine (position 539), and conservative substitutions of all the residues of the hydrophobic core. Interestingly, DNT1 shares more conserved residues with acorn worm Sk-NT than with other NTs (Figure 1B). The DNT1 Cysknot is highly conserved in all sequenced insects, such as fruit fly (*Drosophila*), mosquito (*Anopheles*), and bee (*Apis*) (Figure 2), and conservation outside the Cysknot is also high among all *Drosophila* species.

There is high sequence divergence, particularly outside, but also within the Cysknot among all ancient NTs (Lf-NT, Bf-NT, Sp-NT, Sk-NT, and DNT1). We have attempted phylogenetic analyses of *DNT1* and *spz* compared to all known NTs, including all ancient NTs, as above, using three standard methods (Figure S3). Sequence divergence precludes direct proof of orthology between *DNT1* and vertebrate *NT*s. An ancestral *NT* gene was quite likely the predecessor of *DNT1* in protostomes and *NT*s in deuterostomes.

NTs are secreted proteins with a Cysknot domain, cleaved from pre-pro-precursors, and which dimerise to become functional [46]. Similarly, Spz becomes functional following cleavage to form a Cysknot dimer [40,47–49]. Instead, coagulogen does not dimerise to be functional [41,50]. DNT1 is a 886-amino acid (aa) protein with a 102-aa Cysknot followed by a 286-aa COOH tail, predicted to be secreted and cleaved, possibly at position 498 (Figures 1D, 1E, and S2, predicted cleavage site: FSLSKKR RE; see Text S1). DNT1 is cleaved upstream of the Cysknot in cell culture (Figure 1F), although the putative protease cleaving DNT1 in vivo may be absent in S2 cells. Both recombinant Cysknot and the Cysknot with the COOH extension (Cysknot3-tail) form dimers (Figure 1G), hence they fold correctly upon expression. Thus, DNT1 presents structural, cleavage, and dimerising features of canonical NTs.

### DNT1 Promotes Neuronal Survival and Targeting

The functional characteristics of mature vertebrate NTs are: (1) they are expressed by target cells in limiting amounts; (2) they maintain neuronal survival; and (3) they enable targeting and connectivity. Thus, we asked whether DNT1 satisfies any or all of these criteria.

**Figure 3:**
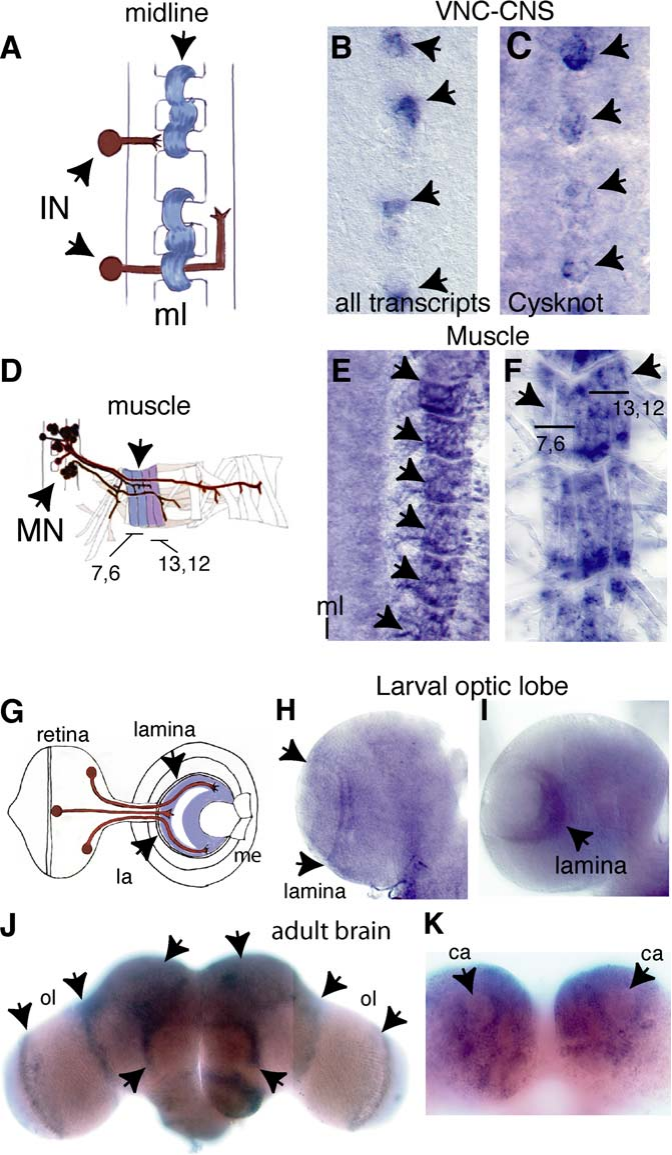
*DNT1* Is Expressed in Target Cells In situ hybridisations to *DNT1* transcripts: (B) probe that detects all isoforms and (C, E, F, and H–K) *DNT1*-Cysknot–specific probe. (A, D, and G) Target tissues expressing *DNT1* (blue), targeting neurons (brown). *DNT1* expression in: (B and C) the embryonic midline (ml) (arrows); (E and F) the embryonic muscle; (F) higher magnification, showing muscles 6, 7, 12, and 13, arrows (A–F), anterior is up; (H and I) the larval lamina (la, arrows); (J and K) the adult brain: (J) dorsal view, *DNT1* is expressed in multiple locations (arrows) including the optic lobes (ol) and central brain; (K) ventral view of central brain, transcripts are present in the cell bodies surrounding the calyx (ca) neuropile (arrows) of the mushroom bodies, site of learning and memory. (K) shows the same specimen as (J). IN, interneuron; me, medulla; MN, motor neuron.

*DNT1* is expressed in target cells throughout development. In the embryo, *DNT1* is expressed at the CNS midline (Figure 3A–3C), the intermediate target for interneurons (the vertebrate floorplate is also an intermediate target that supports neuronal survival [51]), in two lateral CNS cells per hemisegment at stage 17 and in the epidermis (unpublished data), and in the muscles (Figure 3D–3F), the target for motor neurons. In the larva, it is expressed in the lamina (Figure 3G–3I) of the optic lobe, the target for photoreceptor axons. In the adult, it is expressed in the optic lobes and central brain (Figure 3J and 3K), in the site of learning and memory.

In order to analyse the incidence of apoptosis upon loss or gain of function for *DNT1*, apoptotic cells were visualised in vivo with anti-cleaved Caspase-3 (Caspase-3) antibodies, and we developed a computer software programme for the automatic quantification of Caspase-3 stained cells, called DeadEasy (Figure S4, Text S1, and M. G. Forero, J. A. Pennack, A. R. Learte, K. Kato, R. L. Griffiths, et al, unpublished data). Caspase-3 antibodies stain specifically apoptotic cells (Figure S4A); they have the advantage over other methods of not staining necrotic cells, and are extensively used to visualise apoptotic cells in multiple model organisms and in human paradigms (e.g., [13,52–56]). Six or seven trunk segments (depending on stage) of stained embryos are scanned at the confocal microscope throughout the whole thickness of the CNS ventral nerve cord (VNC). A region of interest (ROI) is selected over the VNC for quantification to exclude the epidermis. DeadEasy identifies stained cells in each individual section throughout the VNC and subsequently in 3-D, based on minimum volume and pixel intensity, and produces the total number of cells per VNC. The programme has been verified and validated (see Text S1).

**Figure 4:**
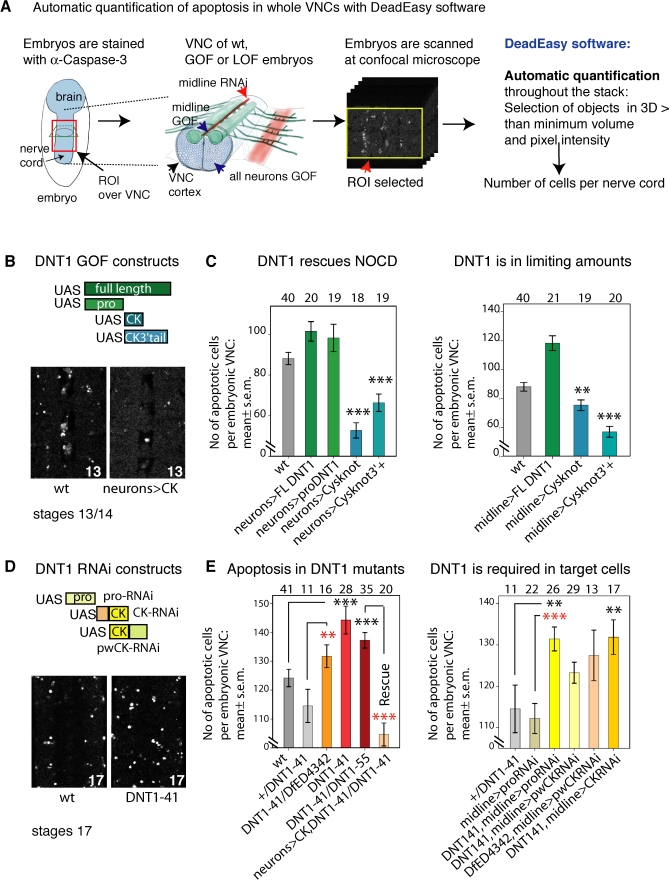
DNT1 Promotes Neuronal Survival in the CNS (A) Embryos are stained with Caspase-3, and six to seven trunk segments of the nerve cord (VNC) are scanned at the confocal microscope (see Text S1), excluding the head and the posterior end. After acquisition, apoptotic cells within a region of interest (ROI) comprising the scanned VNC and excluding the epidermis are quantified automatically. GOF, gain of function; LOF, loss of function; wt, wild type. (B and D) Examples of Caspase-3–stained VNCs. GAL4 targeted expression to: all neurons (*elavGAL4*) and midline (*simGAL4*). Illustration of GOF and RNAi constructs used. (C and E) Results from the automatic quantification of α-Caspase-3–positive cells using DeadEasy software of (stage 13/14 for GOF and stage 17 for LOF). RNAi targeted to the pro-domain or the Cysknot in embryos heterozygote for *DNT1^41^* or *Df(3L)ED4342*. RNAi targeted to the pro-domain knocks-down all DNT1 transcripts, thus all these RNAi constructs reduce cDNA3 levels. Black asterisks are comparisons to wild type, red asterisks to controls. Triple asterisks (***) indicate *p* < 0.001, double asterisks (**) indicate *p* < 0.01, and a single asterisk (*) indicates *p* < 0.05. Numbers over graphs are sample sizes: *n* = number of embryos. For *p*-values and statistics tests, see Text S1. CK, DNT1-Cystine-knot; CK3+, Cysknot3-tail; FL, full-length cDNA3-GFP; pro, pro-domain cDNA1; wt, wild type embryos.

To ask whether DNT1 can rescue naturally occurring cell death (NOCD), we expressed in all neurons (with *elavGAL4*) four forms of the protein: (1) full-length; (2) pro-domain (i.e., cDNA1, lacking the Cysknot, see Text S1); (3) Cysknot; and (4) Cysknot3-tail comprising the Cysknot plus the COOH extension (Figures 1E and 4B). We stained embryos with Caspase-3 (Figure 4A and 4B) and quantified CNS apoptosis in the VNC automatically with DeadEasy software. Expression of either the full-length protein or the pro-domain does not reduce apoptosis levels compared to wild type (Figure 4C). However, expression of either the Cysknot or the Cysknot3-tail results in a significant reduction in apoptosis compared to wild type (Figure 4C). The disparity between the full-length and the cleaved forms is reminiscent of vertebrate NTs [3,46] and of the fact that the cleaved Spz Cysknot is functional when expressed in transgenic flies, whereas full-length Spz is not [47]. Expression of either DNT1 Cysknot or the Cysknot 3-tail at the midline (with *simGAL4*) also reduces significantly apoptosis compared to wild type (Figure 4C), implying that DNT1 is normally present in limiting amounts at this target. These data show that DNT1 can promote cell survival in the embryonic CNS.

**Figure 5:**
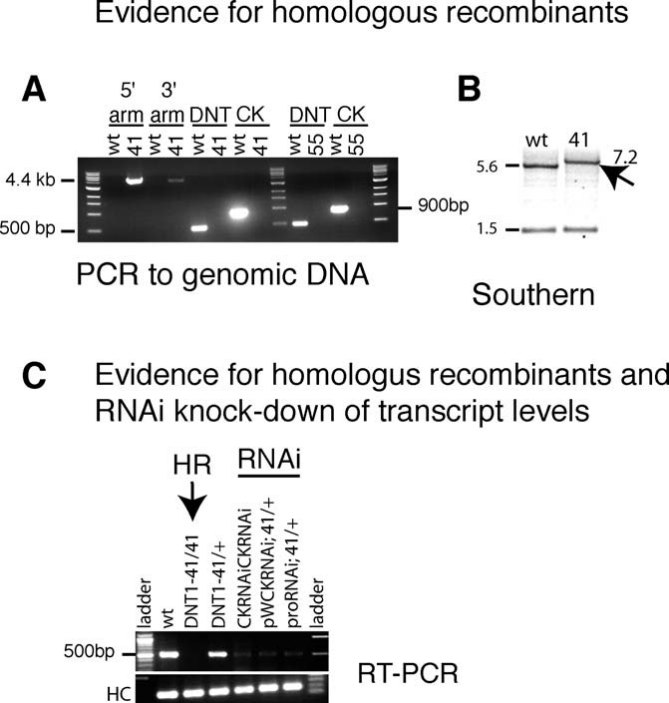
Evidence for Homologous Recombination and RNAi (A and B) Evidence that *DNT1^41^* and *DNT1^55^* are homologous recombinant alleles: (A) PCR evidence that *DNT1^41^* (41) and *DNT1^55^* (55) are null alleles as the coding region of DNT1 has been replaced for that of the *white* gene: 5′ arm: product using primers to *white* and *tie*; 3′ arm: product using primers to *white* and CG13720; DNT: 5′ fragment of coding region. CK, Cysknot domain; wt, wild-type control (*yw* stock). (B) Southern blot evidence that in *DNT1^41^*, the wild-type EcoRI 5.6-kb band shifts to 7.2 kb due to the insertion of *white*. (C) Verification of lack of transcripts in *DNT1* homologous recombinant mutants and knock-down upon targeted RNAi. RT-PCR showing lack of transcripts in *DNT1^41^* null mutant embryos and normal levels of transcripts in *DNT1^41/+^* heterozygous and wild-type embryos (controls). Transcript levels are reduced upon targeted DNT1 RNAi to the midline using simGAL4 for all three RNAi constructs.

To ask whether DNT1 is required to promote cell survival, we generated by homologous recombination genetic null mutant alleles, *DNT1^41^* and *DNT1^55^*, as verified by PCR, Southern blot, and reverse transcriptase PCR (Figures 1C and 5A–5C and Text S1). *DNT1^41^* homozygous mutant flies are viable. In the CNS of homozygous *DNT1^41^* null mutant embryos, apoptosis levels are comparable to wild type at stages 13/14, and there are no axon guidance defects (unpublished data). At stage 17, *DNT1^41^*, *DNT1^41^ /DNT1^55^*, and *DNT1^41^/Df(3L)ED4342* null mutant embryos show a significant increase in apoptosis in the CNS (Figure 4E). To verify that the increase in apoptosis is a direct consequence of loss of *DNT1* function, we expressed the *DNT1* Cysknot in all neurons in embryos mutant for *DNT1* (Figure 4E, rescue). This leads to a significant reduction in apoptosis compared to *DNT1* mutants, confirming that loss of *DNT1* function causes the increase in apoptosis in the mutants.

**Figure 6:**
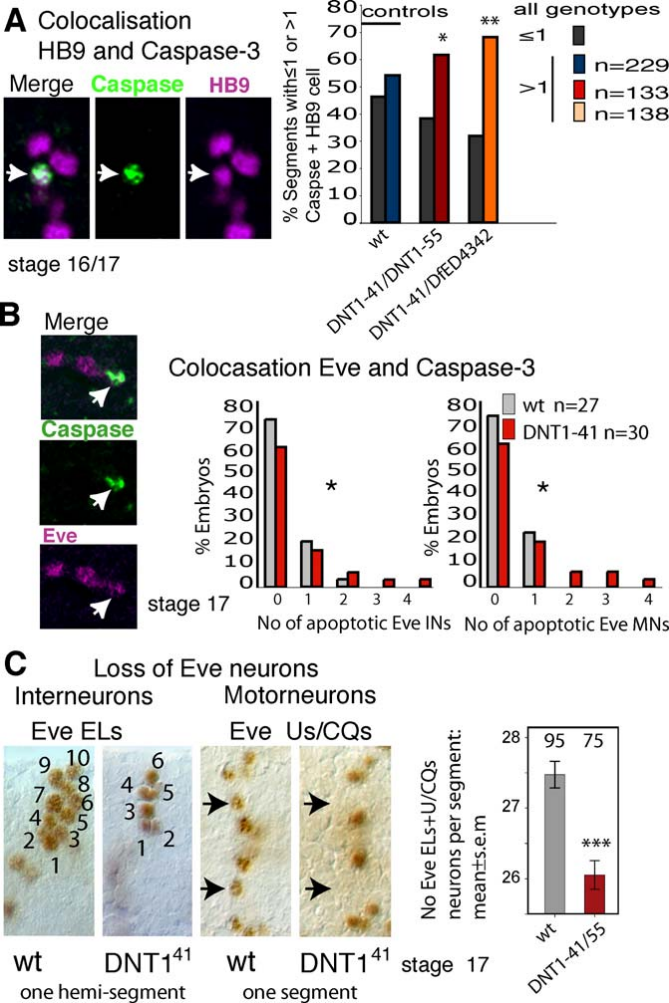
Apoptosis and Loss of Interneurons and Motor Neurons upon *DNT1* Loss of Function Embryonic VNC stained with Caspase-3, and the interneurons and motor neuron markers HB9 and Eve. Quantification of colocalising cells is done manually by looking at each individual section. (A) Colocalisation (arrows, single 0.5-μm section confocal images) of Caspase-3 (green) and HB9 (magenta) (stage 16–17). Graph shows that the percentage of segments with more than one apoptotic neuron increases in *DNT1* mutants, *n* = number of segments. wt, wild type. (B) Colocalisation of Eve (magenta) and Caspase-3 (green), quantification on the right. INs are EL interneurons, MNs are U/CQ motor neurons, *n* = number of embryos. (C) Loss of Eve-positive cells, arrows point at missing cells in *DNT1^41^* mutant embryos. On the right, quantification of Eve-positive cells per segment, numbers over bars: *n* = number of segments. Triple asterisks (***) indicate *p* < 0.001, double asterisks (**) indicate *p* < 0.01, and a single asterisk (*) indicates *p* < 0.05. For *p*-values and statistics tests, see Text S1.

To verify whether the cells dying in excess in the mutants are neurons, we labelled *DNT1* mutant embryos with the neuronal markers anti-HB9 and anti-Eve (as well as Caspase-3), which label complementary sets of motor neurons and interneurons. HB9 stains the majority of the ventrally and laterally projecting motor neurons [57,58]. This corresponds to motor neurons that project via intersegmental nerve (ISN), ISN branch b/d (ISNb/d), segmental nerve branch a (SNa), and SNc, four RP neurons, and a ventral motor neuron, but it does not stain the Eve dorsally projecting motor neurons [57,58]. HB9 is expressed in many interneurons, including serotonergic neurons and a subset of FasII-negative interneurons [58]. Eve-expressing neurons are pCC, fpCC, and EL interneurons and dorsally projecting motor neurons, including aCC, RP2, and the Us/CQs [59]. Colocalisation of Caspase-3 with HB9 increases significantly in *DNT1^41^/DNT1^55^* and *DNT1^41^/Df(3L)ED4342* mutant embryos compared to wild type (Figure 6A). Colocalisation of Caspase-3 and Eve in the EL interneurons and in the U/CQ motor neurons also increases significantly in *DNT1^41^* mutants (Figure 6B). Apoptosis causes cell loss, and there is a significant reduction in the number of Eve-positive neurons in *DNT1^41^* mutants compared to wild type (Figure 6C). These data show that neurons die in excess in the absence of DNT1.

To ask whether DNT1 maintains CNS cell survival nonautonomously from the midline intermediate target, we reduced levels of *DNT1* transcripts containing the Cysknot (CK) by expressing three different *DNT1-RNAi* (RNA interference) sequences in target cells (Figure 4D): *CK-RNAi*, *pwCK-RNAi*, and *pro-domain-RNAi*. The *pro-domain-RNAi* knocks down all *DNT1* transcripts, whereas *CK-RNAi* knocks down only cDNA3. Three different and partially overlapping constructs were used to rule out the contribution of off-target effects to the phenotype. To enhance the specificity and penetrance of RNAi, experiments were carried out in embryos heterozygous for the null allele *DNT1^41^* or for *Df(3L)ED4342* that uncovers the *DNT1* locus. Targeted *DNT1 pro-RNAi*, *pwCK-RNAi*, and *CK-RNAi* restricted to a narrow strip of cells at the CNS midline (with *simGAL4*) increase apoptosis significantly throughout the CNS cortex compared to controls at stage 17 (Figure 4E). Since the shorter cDNA1 does not promote neuronal survival, the increase in apoptosis is due to the knocking down of cDNA3 in all cases. Thus, reducing DNT1 levels at the midline is sufficient to induce apoptosis throughout the VNC. These data, together with the facts that DNT1 mutants have excess apoptosis throughout the VNC despite being expressed in a very small group of cells and DNT1 rescues NOCD when overexpressed at the midline only (Figure 4C), show that DNT1 promotes cell survival nonautonomously in the CNS.

**Figure 7:**
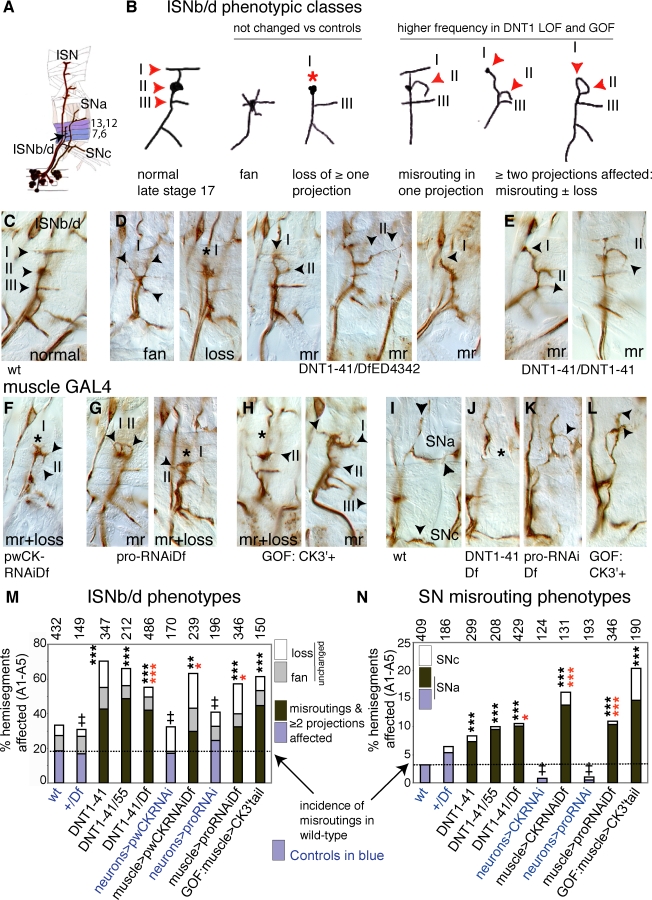
DNT1 Enables Motor-Axon Targeting (A) Projections of motor neurons to the embryonic muscles. (B) Altered DNT1 function results in an increase in ISNb/d misrouting phenotypes. In wild type, there are three stereotypic projections (I, II, and III). Two phenotypes were observed in all genotypes with comparable frequency: “fan” of multiple thin projections originating from II, and “loss” of one or more projections. Misrouting phenotypes, including concomitant effects in two or more projections (e.g., misrouting in two projections, or misrouting in one plus loss of another), are found with higher frequency in experimental genotypes (some examples drawn). (C–H) ISNb/d motor neuron targeting at muscles 7, 6, 13, and 12 visualised with FasII antibodies (brown) in stage 17: (C) wild-type embryos; (D) *DNT1^41^/Df(3L)ED4342* transheterozygote mutants; (E) *DNT1^41^* mutants; (F and G) upon targeted RNAi to the muscle: (F) *24BGAL4* > *pWCysknotRNAi;DfED4342* and (G) *24BGAL4 > pro-RNAi;DfED4342*; (H) upon expression of Cysknot3′tail at the muscle (*24BGAL4 > UASCysknot3′tail*). GOF, gain of function; LOF, loss of function; mr, misrouting; wt, wild type. (I–L) SN projections: (I) wild type; (J) *DNT1^41^/Df(3L)ED4342*; (K) *24BGAL4 > pro-RNAi;DfED4342*; (L) *24BGAL4 > UASCysknot3-tail*. Arrowheads point to projections or misroutings, asterisks to missing projections. Dorsal is up, anterior to the left. (M and N) Quantification of ISNb/d and SN phenotypes: misrouting and effects in two or more projections are shown in brown and for controls in blue. Triple asterisks (***) indicate *p* < 0.001, double asterisks (**) indicate *p* < 0.01, and a single asterisk (*) indicates *p* < 0.05. Black asterisks are comparisons to wild type, red asterisks to controls. Numbers over graphs indicate number of hemisegments. For statistics tests and *p*-values, see Text S1.

To investigate whether DNT1 is required for axonal targeting, we analysed the axonal projections of FasII-positive motor neurons in *DNT1^41^*, *DNT1^41^/DNT1^55^*, and *DNT1^41^/Df(3L)ED4342* mutant embryos and upon *DNT1-CKRNAi* and *DNT1-pro-RNAi* targeted to the muscle (with *24BGAL4*) in embryos heterozygous for *Df(3L)ED4342*. In all cases, there is a significant increase in the incidence of misrouting phenotypes in ISNb/d and SN fascicles compared to wild type, including effects in more than one projection per hemisegment (e.g., misrouting plus loss; Figure 7D–7G, 7J, 7K, 7M, and 7N). To verify the target-dependent origin of these phenotypes, we targeted *DNT1-RNAi* to all neurons as a control. The incidence of axonal phenotypes upon RNAi targeted to all neurons is not significantly different from wild type, whereas it is significantly different from the incidence upon RNAi targeted to the muscle (Figure 7M and 7N). This shows that the phenotypes caused by RNAi targeted to the muscle are due to the loss of DNT1 function in this target. To verify whether targeting to the muscle requires a limited source of DNT1, we overexpressed *DNT1 Cysknot 3-tail* in the muscle. Excess of DNT1 Cysknot3-tail prevents targeting by motor neuron terminals at muscle 6/7 and 12/13 (Figure 7H and 7L–7N). Thus, DNT1 produced by the muscle enables correct motor neuron targeting.

### Neurotrophic Function of Spz and Its Receptor Toll

Given the proposal of an NGF domain in Spz [40,41,43], we asked how Spz relates to the vertebrate NTs. PSI-BLAST search using BDNF and all other vertebrate NTs as query against the *Drosophila* genome fails to identify *spz*. Following the same approach as for DNT1, a PSI-BLAST search using *spz* sequence as a query against the SWISSPROT database produces no significant hit to any NT. *DNT1* and *spz* are paralogs [45], but under the same PSI-BLAST search conditions, DNT1 can be linked to some members of NTs (e.g., fish BDNF), whereas no such link can be established between Spz and any NT. Percentage identity within the Cysknot is higher for DNT1 and BDNF (26.4%) than for Spz and NGF (19%) or than for any other *spz* paralog when compared with NTs. Conservation of *spz* in insects is lower (or absent, e.g., A. gambiae) than that of *DNT1*, but although *DNT1* is not conserved in beetles (*Tribolium*), *spz* is (Figure S5A). These observations suggest that *DNT1* retains the sequence features of an ancient neurotrophin ancestor better than *spz* does.

**Figure 8:**
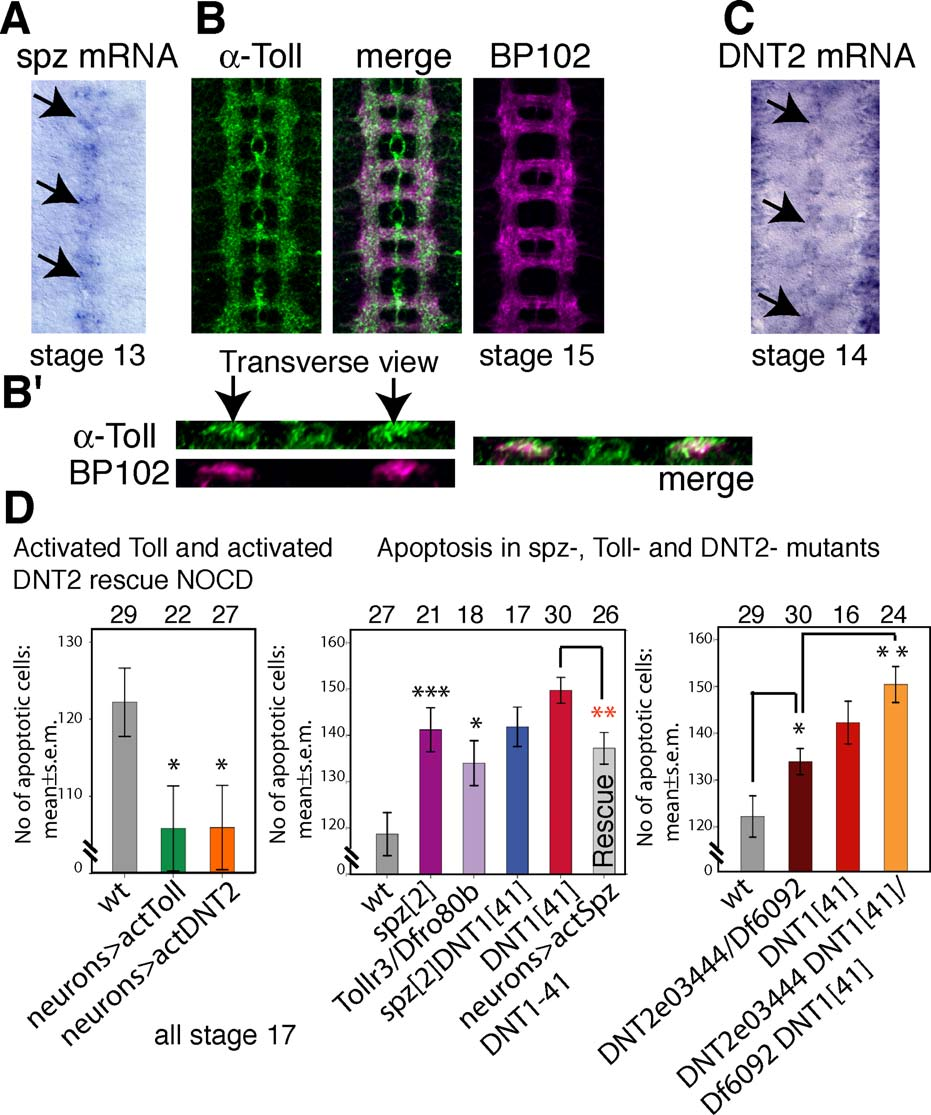
Spz and DNT2 Have Neurotrophic Function (A) In situ hybridisation in a stage 13 embryo showing *spz* transcripts at the CNS midline. (B) Distribution of anti-Toll protein (green) in all CNS neuropile axons, as seen by colocalisation (merge) with the axonal marker BP102 (magenta) at stage 16. Toll is also present in some midline cells. (B′) Transverse sections through the neuropile to show that Toll coincides with the distribution of BP102. (C) In situ hybridisation in a stage 14 embryo showing *DNT2* transcripts at the CNS midline. (D) Automatic quantification of Caspase-3 in loss-of-function and gain-of-function conditions for *spz*, *Toll*, and *DNT2* using DeadEasy software. Expression of activated Toll and DNT2 (*UASDNT2-Cysknot*) in all neurons (with *ElavGAL4*) rescue NOCD: stage 17 embryos. Apoptosis in *spz^2^*, *Toll^r3^/Dfro80b*, and *DNT2^e03444^* mutants: stage 17 embryos. The incidence of apoptosis does not increase in *spz^2^DNT1^41^* double mutants compared to either of the single mutants. Rescue: expression of activated Spz (*UASspz-Cysknot*) in all neurons with *elavGAL4* in *DNT1^41^* null mutant embryos only partially rescues cell death. Apoptosis increases in *DNT1^−^DNT2^−^* double mutants compared to the single mutants. Triple asterisks (***) indicate *p* < 0.001, double asterisks (**) indicate *p* < 0.01, and a single asterisk (*) indicates *p* < 0.05. Numbers over bars are *n* = number of embryos. For *p*-values and statistics tests, see Text S1. wt, wild type.

Nevertheless, Spz still forms a Cysknot [40,41,43] that can be aligned to the NT Cysknot superfamily (Figure 1B). Thus, we next asked whether Spz may have NT function. Spz is expressed at the embryonic CNS midline (Figure 8A), and its receptor, Toll, is in all CNS axons (Figure 8B and 8B′). Expression of activated Toll in all neurons rescues NOCD at stage 17 (but not at stages 13 and 14) (Figure 8D), showing that it can maintain neuronal survival. To ask whether Spz and Toll are required for neuronal survival, we looked at stage 17 embryos where maternal product enables normal early development, as confirmed by the eclosion of homozygous *spz^2 ^* mutant flies. Apoptosis increases in the CNS of *spz^2^* and *Toll^r3^/Df(3R)ro80b* mutant embryos, indicating that both Spz and Toll are required for neuronal survival (Figure 8D). Altogether, these data show that *spz* also has neurotrophic function, but it is weaker than DNT1. DNT1 and Spz do not seem to play fully redundant roles. Apoptosis does not increase further in *spz^2^ DNT1^41^* double mutants, and expression of activated *spz* in *DNT1^41^* mutant embryos rescues apoptosis slightly, but it does not rescue the *DNT1^41^* mutant phenotype (Figure 8D). We cannot rule out the possibility that DNT1 may rescue the *spz* mutant phenotype. This indicates that DNT1 and Spz may promote the survival of distinct subsets of neurons.

### The Functions of DNT1 and Spz Are Specific to Neuronal Modality

**Figure 9:**
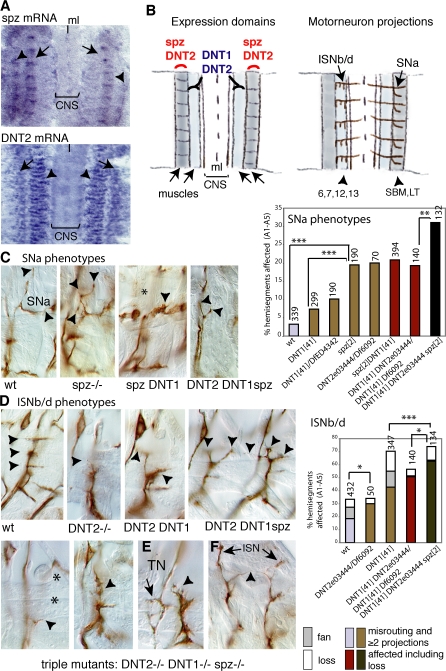
Complementary and Synergistic Functions of DNTs in Targeting (A) Distribution of *spz* mRNA in two lateral bands below the epidermis, at the location of the segment boundary muscle (SBM) and lateral transverse (LT) muscles. Below, distribution of *DNT2* transcripts in two domains: over muscles 6, 7, 12, and 13 overlapping with *DNT1* expression (arrowheads) and a second domain overlapping with *spz* expression (arrow). *DNT2* is also expressed in other muscles (not shown). ml, midline. (B) Diagrams illustrating the complementary expression domains of *DNT1*, *DNT2*, and *Spz.* On the far right, the motor neuron projections from ISNb/d are shown on the left half, and the projections from SN are shown on the right half for clarity. ISNb/d project to muscles 6, 7, 12, and 13 that express *DNT1* and *DNT2*; SNa project to SBM and LT muscles that express *spz* and *DNT2.* (C and D) Quantifications of axonal phenotypes are colour coded by genotype: controls in blue; single mutants in gold; double mutants in red; and triple mutants in dark brown. (C) Axonal phenotypes of the SNa motor axons, quantification on the right. There is a significantly higher percentage of hemisegments with axonal misrouting defects in the SNa projections in *spz^2^* , *spz^2^DNT1^41^* double, and *DNT2^eo3444^DNT1^41^ spz^2^* triple mutants than in *DNT1^41^* mutants. No significant increase in axonal misroutings was found for ISNb/d projections in *spz^2^* mutants. (D) ISNb/d phenotypes in *DNT2^ eo3444^/Df6092*, *DNT2^eo3444^DNT1^41^* double-, and *DNT2^eo3444^DNT1^41^ spz^2^* triple-mutant embryos. Both the frequency and severity of misrouting phenotypes (arrowheads) increase in the double and triple mutants, including loss of all projections (asterisks). Two hemisegments are shown for the triple mutant. (E and F) Dramatic misroutings in *DNT2^eo3444^DNT1^41^ spz^2^* triple-mutant embryos in (E) the transverse nerve (TN) (which, however, do not increase in frequency) and (F) ISN (penetrance in triple mutants 12.7% vs. 0%–1.8% in single and double mutants, respectively). Triple asterisks (***) indicate *p* < 0.001, double asterisks (**) indicate *p* < 0.01, and a single asterisk (*) indicates *p* < 0.05. Numbers over bars are *n* = number of hemisegments. (C–F) all stage 17. All axonal images show projections in one hemisegment, except for far right in (D and F) that show two. For *p*-values and statistic tests, see Text S1.

*spz* is also expressed in bands along the location of embryonic lateral muscles (Figure 9A), in a complementary pattern to that of *DNT1* in muscles (Figures 3E and 9B), suggesting that they may aid targeting by different axonal projections. Loss of Spz function affects predominantly targeting by the SNa motor axons (Figure 9C). Loss of DNT1 affects ISNb/d projections more severely that SNa projections (Figure 7M and 7N). The SNa axonal phenotype of double-mutant embryos lacking both DNT1 and Spz functions (*spz^2^ DNT1^41^*) is epistatic to *spz* (Figure 9C). Altogether, these observations suggest that the targeting functions of DNT1 and Spz depend on neuronal modality.

### A Third *Drosophila* Neurotrophin, DNT2

*DNT1* is closer to *spz* and *spz5 (CG9972)* than to the other paralogs [45] (see Table S2). Structure-based alignment using FUGUE reveals that DNT1/Spz2, Spz, and Spz5 are more closely related to each other and to human NTs, whereas Spz3 *(CG7104)* and Spz6 *(CG9196)* are less closely related to vertebrates NTs (Table S2). Spz5 is very highly conserved amongst insects (Figure S5B).

The Cysknots of Spz3, Spz4 (*GC14928*), and Spz6 differ from the canonical NT Cysknot: Spz3 and Spz4 have two extra cysteines and the Spz6 Cysknot lacks two of the conserved cysteines and has three extra ones in unusual locations. The Cysknots of *Spz6* and *Spz4* also differ from the rest in that they lack a conserved intron [45]. Furthermore, Spz4 is closest to coagulogen (29% identity), while also being closer to Spz3 (51% identity) than to other paralogs, and its expression is up-regulated upon immune challenge [45]. Thus, the six *spz* paralogs fall into two groups: one formed by *DNT1/spz2*, *spz*, and *spz5*, and the other formed by *spz3*, *spz4*, and *spz6*. Nevertheless, at least five of the six paralogs are expressed in the nervous system. There are no mutants available for *spz3*, -*4*, and -*6*. Structural considerations suggest that Spz5 could also have neurotrophic function.

To investigate whether *spz5* has neurotrophic function we first visualised its expression pattern. *spz5* is expressed at the embryonic CNS midline (Figure 8C), in muscles (Figure 9A), in the epidermis (unpublished data), and in the embryonic head peripheral nervous system (PNS) (unpublished data). To ask whether Spz5 can maintain neuronal survival, we expressed the Cysknot domain of *spz5* (*UASDNT2 CK*) in all neurons (with *ElavGAL4*), which rescues NOCD (at stage 17) (Figure 8D). To ask whether loss of *spz5* function affects neuronal survival, we used the only available mutant allele—*spz5^e03444^*—and deficiency *Df(3L)Exel6092* uncovering the *spz5* locus. There is an increase in apoptosis in *spz5^e03444^*/*Df(3L)Exel6092* transheterozygous mutant embryos (Figure 8D). Altogether, these data mean that Spz5 maintains neuronal survival in the embryonic CNS. Loss of both *DNT1* and *spz5* in double-mutant embryos results in an increase in apoptosis compared to each of the single mutants (Figure 9D), revealing redundant or complementary functions in the control of neuronal survival. Thus, we rename *spz5* (*CG9972*) as *DNT2*.

In the muscle, the expression of *DNT2* overlaps with that of both *DNT1* (in muscles 6, 7, 12, and 13) and *spz* (in SBM, LT lateral muscles). Both ISNb/d and SNa projections are mildly affected in *DNT2^e03444^/Df(3L)exel6092* mutant embryos. The penetrance of ISNb/d targeting defects increases in *DNT1 DNT2* double-mutant embryos, although not significantly (genotype: *DNT2^e03444^ DNT1^41^/Df(3L)Exel6092 DNT1^41^*) (Figure 9D). The penetrance of both SNa and ISNb/d targeting defects increases in *DNT2^e03444^ DNT1^41^spz^2^* triple-mutant embryos, compared to the double or single mutants (Figure 9D). In the triple mutants, misrouting phenotypes can be very dramatic, and there are cases of loss of all ISNb/d motor axons (not seen in single mutants) (Figure 9D, far left). Misrouting of the transverse nerve (TN) can be very dramatic in triple mutants (Figure 9E), although milder effects in this nerve occur with comparable penetrance in all genotypes (∼10%). Misroutings of ISN are negligible in single and double mutants, but they increase and can be dramatic in triple-mutant embryos (12.7%, Figure 9F). These findings indicate that there is a synergistic interaction between DNT1, Spz, and DNT2 in targeting, suggestive of redundant functions in this context.

Synergism between the DNTs is further revealed by the effects of these mutations in viability. Whereas both *DNT1^41^* and *DNT2^e03444^* mutants are viable and fertile, viability is somewhat affected in *DNT1^41^ DNT2^ e03444^* double mutants (Table S3): in homozygosis, *DNT1^41^ DNT2^ e03444^* flies are viable (although some larval lethality, as well as when in trans over *DNT1^41^ Df(3L)6092*, was observed), but *DNT1^41^ DNT2^ e03444^/TM6B* flies do not produce homozygous progeny at 18 °C, suggesting unsuccessful larval competition. Whereas homozygous *spz^2^* flies can eclose as adults, the *DNT1^41^ spz^2^* double and triple mutants are completely lethal (100% penetrance). This suggests that DNT1, Spz, and DNT2 play redundant functions for viability.

### Adult Flies Lacking DNT1 and DNT2 or Spz Have Locomotion Deficits

Neurotrophin mutant mice display abnormal locomotion [60–63].To ask whether DNT mutant flies move normally, we tested the ability of adult flies to climb over the rim of a Petri dish and walk along it—something wild-type flies do without difficulty and without falling off (Video S1). *DNT1^41^ DNT^e03444^* and *DNT1^41^ DNT2^e03444^/DNT1^41^ Df(3L)Exel6092* double-mutant flies display a range of phenotypes (Table S4) including: inability to estimate the location of the rim (Videos S2, S3, and S6), falling off (Video S3 and S5), sluggishness (Video S4), inability to climb (Video S6), slow, uncoordinated movements (Video S7), and wobbling (Video S8); *Spz^2^* mutant flies can barely walk (Video S9). These phenotypes may be due to abnormal targeting or muscle structure or function or synaptic activity. They suggest that an involvement of DNTs in higher neuronal functions is a possibility.

## Discussion

We provide bioinformatic and functional evidence that DNT1 is a NT, and it forms a family with at least two further *Drosophila* NT members, Spz and DNT2.

Neurotrophic factors had been anticipated in *Drosophila* but not previously found. We have identified DNT1 and shown that it satisfies the criteria to be a NT superfamily member. First, DNT1 was identified by sequence homology to NTs through sequence-based bioinformatic searches. Sequence identity to NTs is not high and is restricted to the Cysknot domain. However, this conservation is sufficient to ensure the structural features of a NT Cysknot. Second, DNT1 is structurally a NT superfamily member. DNT1 is predicted to be secreted, it is cleaved and forms a NT-Cysknot, which dimerises to become functional. A structure-based alignment shows conservation of all the residues relevant to forming the Cysknot, not only between DNT1, vertebrate, and human NTs, but also including the ancient NTs from *Amphioxus*, sea urchin, and acorn worm. Third, DNT1 functions like a canonical NT: loss of DNT1 function results in increased neuronal apoptosis, gain of DNT1 function rescues NOCD, and interfering with DNT1 function affects targeting by embryonic motor axons. In the CNS, neuronal survival depends on DNT1 produced in limiting amounts from the midline intermediate target. Targeting by the motor axons requires DNT1 at the muscle. The high conservation of DNT1 in insects supports its functional relevance. Adult flies mutant for *spz* or double mutant for *DNT1* and *DNT2* have distinct locomotion deficits. *DNT1* is expressed in the brain, in the centres for learning and memory, suggesting possible higher neuronal functions.

### A NT Family in *Drosophila* Formed by DNT1, DNT2, and Spz

Previous reports had revealed an NGF domain in Spz and biochemical evidence supports a similar mechanism of activation for Spz and the vertebrate NTs [40,47,49]. A theoretical structural analysis of Spz had shown that Spz forms a NT Cysknot [41,43] . These are features also found in DNT1. However, when we carried out bioinformatic searches for Spz, a relationship between Spz and the NTs could not be established. Sequence identity between Spz and NGF is lower than for DNT1 and BDNF. Spz is also less conserved in insects than DNT1 is. The sequence of Spz is more diverged from the vertebrate NTs than DNT1 is. Nevertheless, Spz, together with Toll, also plays neurotrophic functions.

Structural analysis of the *spz* paralogs indicates that DNT1, Spz, and Spz5 are more closely related to each other and to the NTs, whereas Spz3, Spz4, and Spz6 are highly diverged. We cannot at this stage rule out the possibility that Spz3, Spz4, and Spz6 may also play functions in the nervous system. Spz5 is structurally close to the NTs and very highly conserved in insects. We have shown that Spz5/DNT2 has neurotrophic functions, as it rescues NOCD, and loss of Spz5/DNT2 function results in increased CNS apoptosis and axon targeting errors. We have renamed *spz5* as *DNT2*. Thus, there is a NT family in *Drosophila* formed of at least DNT1/Spz2, DNT2/Spz5, and Spz.

### Neurotrophin Superfamily: High Sequence Divergence in the Invertebrate NTs

**Figure 10:**
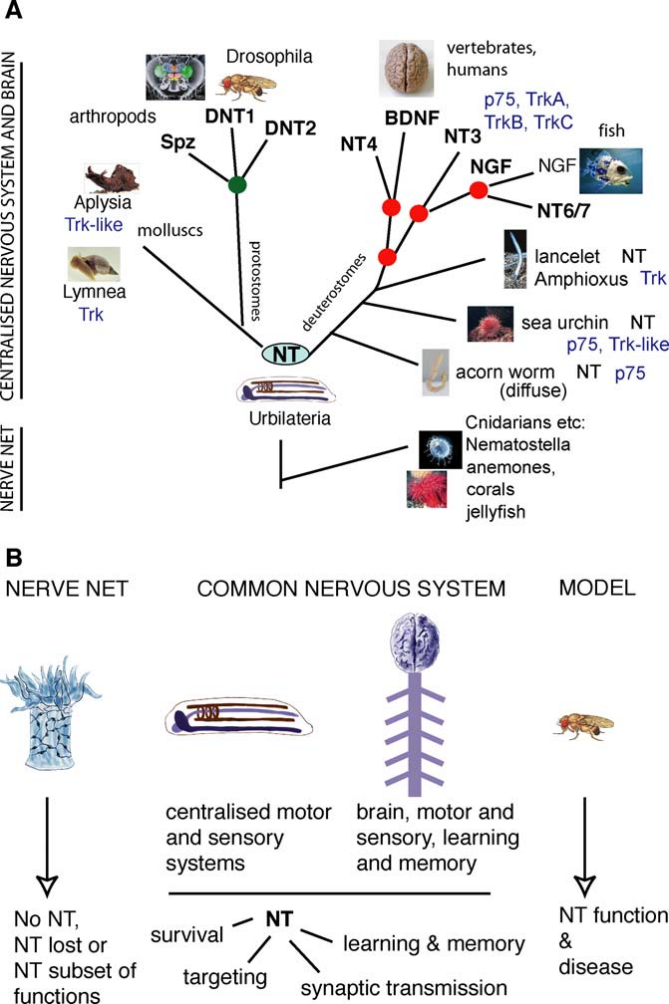
A Common Origin for the NT Superfamily Underlies Nervous System Structure and Function (A) NT superfamily members identified in protostomes (*Drosophila*) and deuterostomes (vertebrates, *Amphioxus*, sea urchin, and acorn worm) imply that NTs were present in *Urbilateria*, their common ancestor. A protostomian *NT* gene would have duplicated to give rise to *DNT1*, *DNT2*, and *spz* in insects or perhaps earlier. A chordate *NT* duplicated twice to give rise to the four vertebrate NTs, and the *NGF* ortholog duplicated again in fish to result in *NT6/7.* Identified Trk and p75 receptors are also shown; Trk-like receptors lack some extracellular domains. NT receptors and signalling mechanisms may have diversified through evolution. Annelids, flatworms, nematodes, and tunicates are not shown, see Figure S1. (B) Hypothesis that the NTs are required in all centralised nervous systems to link structure and function. NTs are also present at least in acorn worm with a nerve net, a diffuse nervous system, where NT may play a subset of functions, suggesting that NTs could also be present in lower animals (e.g., Cnidarians). *Drosophila* can be used as a model system for NT-related studies.

Orthologs are genes related by ancestry. The identification of *DNT1* by sequence homology to *BDNF* does not mean that *DNT1* is a *BDNF* ortholog. *BDNF* resulted from the duplication of an ancestral vertebrate *NT*, thus a relationship between *DNT1* and vertebrate *NT*s goes back to an ancestral *NT* (Figure 10A). Consistently, *DNT1* and *Spz* are more closely related to *Sk-NT* from acorn worm. The sequence relatedness between *DNT1* and the *NTs* is unlikely to be due to convergence since it was found using three independent types of searches, including a structure-based search, and confirmed with two types of reverse searches, and biochemical features and function are also conserved. Direct proof that *DNT1*, *DNT2*, and *spz* are general *NT* orthologs cannot be obtained. High sequence divergence amongst all invertebrate *NTs* precludes the phylogenies to resolve. The same conclusion had been reached for the analysis of ancient deuterostomian *NT*s [38]. Our phylogenetic analyses of *DNT1* and *spz* compared to all known *NT*s, revealed interesting features: first, the invertebrate deuterostomian NTs are closer to DNT1 and Spz than the vertebrate NT. Second, amongst those, acorn worm NT (Sk-NT) is the closest to DNT1 and Spz. Third, two other protein families contain Cysknots, TGFβ and PDGF, but these Cysknots differ from that of NTs. The Cysknot in DNT1 and Spz [41] is unequivocally closer to the NT Cysknot. The most parsimonious explanation (Figure 10A) is that an ancestral *NT* gene present in *Urbilateria* (the presumed common ancestor of all bilateral organisms) gave rise to the *NT*s in deuterostomes and in protostomes. The deuterostome *NT* duplicated twice to give rise to *BDNF*, *NGF*, *NT3*, and *NT4* in vertebrates, and the protostome ancestor duplicated more than once to generate at least *DNT1*, *DNT2*, and *spz*, while sequences diverged, retaining the structural features of the NT Cysknot that enabled function.

A similar scenario is encountered in the tumour necrosis factor (TNF) superfamily, in which sequence similarity and identity between TNF members is restricted to the TNF homology domain where it is also low (19%–30%), but they are nevertheless considered members of a protein superfamily based on structural and functional conservation [64]. Thus, deuterostomian invertebrate NTs (Bf-NT, Sp-NT, and Sk-NT) belong to the NT superfamily based on sequence similarity in the Cysknot [34,38], and we show that DNT1, DNT2, and Spz belong to the NT superfamily based on sequence, structural, and functional criteria.

### Why Had NTs Been Missing in *Drosophila*?

It had long been thought that NTs were missing from the *Drosophila* genome [42,65–68]. A similarity between Spz and NGF had been previously proposed [40,41] but remained controversial. First, structural considerations had also revealed a similarity between Spz and horseshoe crab coagulogen [41], involved in the blood-clotting cascade. However, this study [41] did not use FUGUE, which was developed later to infer structural relationships between distantly related proteins [44]. A later study confirmed that Spz belongs to the NT superfamily [43]. Our phylogenetic analysis does not resolve coagulogen as sufficiently distinct from DNT1, Spz, or the NTs. The Toll signalling cassette is conserved in horseshoe crab, including a Toll receptor and the downstream target NFκB [37,69]. Although it is unknown whether coagulogen may also have NT function in the horseshoe crab CNS, it is an intriguing possibility. We show here that FUGUE analysis comparing DNT1 to all proteins of known structure reveals a closer relationship of DNT1 to vertebrate NTs than to coagulogen.

Second, an initial comparison of the sequenced human and *Drosophila* genomes with BLAST reported that there were no NTs in *Drosophila* [42,68]. However, this simple BLAST missed 30% of the *Drosophila* genes and would have missed any proteins with structural conservation despite sequence divergence. In fact, a recent report has reiterated the relationship of Spz to the NT superfamily [43]. We identified DNT1 using searches optimised for distantly related sequences, PSI-BLAST and FUGUE. In PSI-BLAST sequence searches, carp BDNF reveals sequence relatedness of DNT1 to NTs. Reverse BLAST and PSI-BLAST reveal similarity of DNT1 to BDNF from multiple fish species and humans. Structure-based searches with FUGUE demonstrate that DNT1 is structurally related to human BDNF, NGF, NT3, and NT4. Thus, DNT1 retains the features of all four human NTs. Thus, there is high sequence divergence among the NTs that nevertheless retain the functional Cysknot.

### Similarities and Differences in NT and DNT Functions in the CNS

The neurotrophic theory originally proposed that NTs promote neuronal survival in a target-dependent manner [1], although NTs can also promote neuronal survival prior to innervation and in autocrine and paracrine manners [4,70]. Important evidence that vertebrate NTs promote neuronal (and glial) survival was the finding that exogenous application of NTs rescues neurons (and glia) from NOCD, both in cell culture and in vivo [71–82]. We find that expressing *DNT1* either in all CNS neurons or at the midline can rescue NOCD in vivo. Expressing *DNT2* or activated Toll in all CNS neurons also rescues NOCD. These findings indicate that, like in vertebrates, the DNTs can promote cell survival. The prosurvival functions of the DNTs are nonautonomous as the three DNTs are expressed virtually only at the CNS midline, but in the mutants, apoptosis is induced throughout the VNC; DNT1-RNAi targeted to the midline induces apoptosis throughout the VNC, and overexpression of *DNT1* only at the midline rescues NOCD throughout the VNC.

Loss of vertebrate NTs in individual mouse NT knockouts or their receptors affect the CNS very weakly, and do not generally cause an increase in CNS apoptosis [60–63,83–90]. Loss of *DNT1*, *spz*, *Toll*, or *DNT2* function does not cause massive CNS neuronal death either. Nevertheless, apoptosis increases significantly in the embryonic CNS in all *DNT* mutants. The dying cells are at least partly HB9 and Eve neurons. We did not find significant apoptosis phenotypes in *DNT1* mutants or upon gain of function in the developing retina (unpublished data).

Vertebrate NTs play partially redundant functions [60,61,63,72,83–86]: some can substitute for one another to rescue apoptosis in mutants, and in multiple knock-out combinations, e.g., *BDNF^−/−^NT3^−/−^NT4^−/−^* or *TrkB^−/−^TrkC^−/−^*, a 20% reduction in motor neurons and a dramatic increase in brain apoptosis, respectively, were observed compared to single mutants. The DNTs play redundant roles in the embryonic CNS in some, but not all, contexts. Expression of activated *spz* in *DNT1^41^* mutant embryos is not sufficient to fully rescue apoptosis (however, we have not tested the reciprocal experiment), but apoptosis increases in *DNT1^−/−^ DNT2^−/−^* double mutants, indicating redundancy between DNT1 and DNT2 for cell survival.

Vertebrate NT function depends on neuronal modality: different neurons require different NTs for survival, and increases in apoptosis in the brain were observed when looking at specific neuronal types (e.g., parvalbumin-positive neurons in *BDNF* knock-out mice) [60,72,84,91]. In *DNT1* mutants, we observe an increase in apoptosis of HB9- and Eve-positive neurons, and loss of Eve neurons. Neuronal modality differences are revealed in the targeting by motor axons (see below). Alterations in DNT1 function affect primarily ISNb/d motor axons, whereas loss of Spz function affects SNa motor axons, correlating with complementary domains of *spz* and *DNT1* expression in different subsets of muscles.

Locomotion deficits and/or lethality are a further feature of *NT* knock-out mice [60–63]. In fruit flies, some double-mutant combinations of the DNTs and triple mutants die during embryogenesis. *DNT1 DNT2* double-mutant and *spz^2^* mutant viable adult flies have distinct locomotion and/or behavioural deficits. Locomotion defects can reflect proprioception or muscle or synaptic problems. NTs play roles in synaptic plasticity, LTP, and behaviour, and altered NT function causes psychiatric and cognitive disorders in humans [2,3]. At least *DNT1* is expressed in the adult central brain in the centres controlling learning and memory. Perhaps the DNTs are involved in higher neuronal functions.

*DNT1* produces two types of transcripts: the longer contain the Cysknot domain (cDNA3), and shorter ones (cDNA 1, cDNA2, and cDNA4) comprise only most of the pro-domain. We have shown that expression of the shorter isoform does not rescue apoptosis, rather it (and the full-length protein) may increase it (see Figure 4C). This is reminiscent of the opposite functions of the mature and full-length vertebrate NTs in the control of neuronal survival and death, respectively [3], and of the fact that in transgenic flies, full-length Spz is not functional in immunity, whereas the cleaved Cysknot is [47,48]. We do not know whether the shorter DNT1 isoforms play other roles, but conceivably they may modulate the function of mature DNT1, as the pro-domain of *spz* can inhibit signalling by the Spz-Cysknot [43,92].

### Similarities and Differences in NT and DNT Functions in the PNS and Motor Neurons

Loss of vertebrate NTs severely affects the PNS, and rather weakly affects the motor neurons [60–63,83–88,93]. Virtually all vertebrate PNS neurons require NTs for survival. In *Drosophila*, the effect of *DNT1* mutations in the embryonic PNS is milder than in the CNS (unpublished data). Exogenous application of NTs can rescue vertebrate motor neuron survival [76–78], but loss of individual vertebrate NTs does not induce motor neuron apoptosis [61–63,88]. Only 20%–30% of motor neurons die in triple knock-out mice lacking multiple NTs or all Trk receptors [85,93]. In fact, the main trophic factor maintaining vertebrate motor neuron survival is GDNF, which does not belong to the NT superfamily (e.g., [94]). Motor neurons are not produced in vast excess in *Drosophila*, but there is motor neuron apoptosis in normal embryos, as detected with the motor neuron markers HB9 and Eve, although the underlying cause is not known [13]. We observe a significant increase in HB9 neuronal apoptosis in *DNT1* mutant embryos compared to wild type (although HB9 also labels interneurons). Loss of Eve motor neurons is also observed in *DNT1* mutants, as well as loss of all the FasII-positive ISNb/d axons in triple-mutant embryos. It has previously been reported that RP motor neurons can be missing in *Toll* mutant embryos, although this could reflect an autocrine function [95]. We have not been able to conclusively determine whether motor neuron death in *DNT1* and triple mutants is due to the target-derived function of DNTs in the muscle, or an autocrine/paracrine requirement in the motor neurons. Expression of *DNT*s at the midline could influence the motor neurons within the CNS. Abundant evidence indicates that motor neurons live and function well in the absence of the muscle target in *Drosophila* [96]. For instance, upon genetic elimination or surgical ablation of the muscle [97–99] and in the absence of muscle-derived signals [100], motor neurons grow towards the muscle but fail to target or target to ectopic sites. In normal embryos and larvae, the projection patterns of motor neurons is very stereotypic [96,101,102]. Accordingly, it would appear that motor neuron survival may not depend on the target muscle in *Drosophila* embryos and larvae.

Vertebrate NTs influence muscle innervation by motor neurons [103]. In *Drosophila*, the existence of a muscle-derived sprout-promoting factor to which *Toll-*expressing motor neurons would respond had been anticipated [95]. We show that a target-derived function of DNTs in the muscle is required for guidance and targeting by motor axons. Loss of function for all three DNTs, as well as gain of DNT1 function, disrupts axon guidance and targeting by motor axons. The domains of expression of *DNT1* and *spz* in the muscles are complementary, and both overlap that of DNT2. Consistently, *DNT1* and *spz*, together with *DNT2*, affect targeting by complementary sets of motor axons, and the triple mutants have dramatic defects in all motor neuron projections (see above).

The larval neuromuscular junction (NMJ) offers the most amenable synapse in *Drosophila*. There is abundant evidence of synaptic plasticity at the NMJ [104,105]. However, so far, the identification of the responsible retrograde signals has been rather scarce [105–108]. The identification of the muscle-derived secreted DNTs is promising in this context.

All three DNTs are expressed at the CNS midline and in the muscles. At least the Spz receptor *Toll* is expressed transiently in the muscle; *Toll* and *spz* mutants have muscle defects, and Toll is involved in motor neuron synaptogenesis [95,109], although some of the *Toll* mutant muscle defects may be due to nonautonomous effects [110]. We have also observed muscle defects in *spz* mutants and most severely in the triple mutants. However, targeting errors were also observed in the presence of normal muscle patterns (see Figure S6), indicating that targeting and putative muscle functions can be dissociated. We cannot rule out the possibility that DNTs may play roles in midline-derived glia or neurons, including motor neurons, or in the muscles. Interestingly, vertebrate NTs also have functions in the muscle [111].

### Evolution of NT Receptor and Signalling

Signalling by DNT1 and DNT2 may not necessarily proceed by binding canonical vertebrate-like Trk and p75 receptors. Ligand and receptor pairs do not necessarily coevolve [34,112]. For instance, Toll-like receptors are highly conserved, but bind very different ligand types in flies and vertebrates [113]. DNT1 and DNT2 may bind yet-unidentified Trk and p75 homologs in *Drosophila* or other receptors that activate equivalent signalling pathways and result in equivalent cellular, neurotrophic responses. Trk homologs were originally reported in *Drosophila* and subsequently showed not to belong to the Trk family [39]. However, a Trk homolog has been found in the protostome mollusc *Lymnea* [36,39], suggesting that either Trks may have been lost in *Drosophila* or not found. Trk receptors are modular, thus exon shuffling during evolution could have led to the separation of domains into different proteins while retaining function [34,112]. Consistently, an intracellular Trk-like tyrosine kinase domain has been found in *Aplysia* in a receptor, ApTrk, with an extracellular domain unrelated to the Trks [35]. The converse situation is conceivable.

DNT1 may bind a receptor tyrosine kinase, or a TNFR-like receptor (as p75 is), or resembling Spz, a Toll-like receptor, or, as with vertebrate NTs, DNT1 may be a promiscuous ligand binding multiple receptor types. As with vertebrate NT receptors, binding to one receptor type may result also in interactions with other receptors that alter cellular outcomes depending on context. There is a TNF receptor and multiple Toll-like receptors in *Drosophila* [114]. Signalling by Toll and mammalian Toll-like receptors underlies innate immunity [115], and it is an ancient pathway present also in the cnidarian *Nematostella* and in Caenorhabditis elegans. Vertebrate NTs are also involved in immunity. Perhaps Toll signalling is an ancient mechanism underlying the functions of both the nervous and immune systems. Interestingly, the extracellular domain of Toll resembles that of Trk receptors (with the unusual combination of Leu-rich repeats and cysteine repeats), and intracellularly, Toll activates a downstream signalling pathway very similar to that of p75, resulting in the activation of NFκB [34]. Our data indicate that the evolutionary trajectory of neurotrophin signalling in arthropods travelled through—although may not be restricted to—Toll. DNTs may also bind other receptor types.

Toll, p75 and the TNFR family are more ancient than the Trks [30]. *Drosophila* Spz/Toll, and vertebrate Toll-related, p75 and TNFR receptors signal through NFκB (promoting cell survival) and c-Jun (promoting cell death) [115]. Vertebrate Toll-like–related receptors also activate MAPKinases [115], and p75 also activates AKT [30]. These pathways are compatible with the neurotrophic functions of DNT1, DNT2, and Spz. NFκB is also involved in synapse formation, synaptic plasticity, learning, and memory, and alterations in NFκB function also lead to psychiatric conditions [116,117]. Inhibition of NFκB signalling in crabs (protostome arthropods like flies) leads to deficits in learning and memory, functions traditionally assigned to NTs [118]. Conceivably, also higher functions of DNTs may be controlled by NFκB.

Our findings and those of others [33–36,38,41,45] suggest that the evolution of neurotrophin signalling may have resulted in diversification of receptors and/or downstream signalling pathways.

### Ancestral Origin of NTs in Animals

We have not found DNT1 sequences in the snail *Aplysia* (see Text S1). This could mean that NTs appeared independently in deuterostomes and insects, and their similarity is due to convergence. However, it is equally possible that structure and function were conserved despite high sequence divergence, that the sequences have not been found yet, or that NT were lost from some or many animals. A Trk-like tyrosine kinase domain has been found in *Aplysia*, ApTrk [35], and a bona fide Trk ortholog in another snail, *Lymnea*, suggesting that the NT signalling pathway is present in molluscs. Our unsuccessful search in *Aplysia* is likely due to incomplete genome sequence and expressed sequence tag (EST) collection [119].

If a NT was present in *Urbilateria* (Figure 10A), then NTs may be important in the nervous system development and function of all animals with a centralised nervous system or brain. What about simpler animals such as anemones and corals, which do not have a centralised nervous system, but a diffuse, nerve net (Figure 10A and 10B)? To ask this, we searched for NTs in a cnidarian, *Nematostella*, but we did not find a DNT1 homolog. Sequence divergence and/or incomplete EST database may have also prevented the identification of NT sequences in *Nematostella*. Orthologs of Toll and downstream targets of Toll, p75, and Trk receptors, such as NFκB, MAPKinase, and ERK, are all present in *Nematostella* [120]. Alternatively, NTs may have originated in *Urbilateria* and are absent from simpler animals, or perhaps a preexisting NT may have been lost in *Nematostella* and other cnidarians (just as NTs were lost in the deuterostome *Ciona* [38]), as extensive gene loss is known to have occurred in cnidarians [121]. Consistently with the view that elaborations of neurotrophin signalling underlie brain complexity, perhaps the diffuse net structure of the cnidarian nervous system does not require neurotrophin signalling, resulting in their loss. However, the acorn worm also has a diffuse, nerve net nervous system, and it has a NT and p75 receptor. This suggests that NTs may also be present in other animals with a nerve net, where they may have a subset of functions (e.g., axon guidance, connectivity, or synaptic functions).

### Conclusion and Implications

Our data suggest that a NT was most likely present in *Urbilateria*, the common ancestors of all bilateral organisms—protostomes and deuterostomes (Figure 10A)—it duplicated independently in vertebrates and invertebrates, and NTs were retained in organisms with a centralised nervous system and/or brain. NTs may be more ancient and have been either retained or lost in animals with diffuse neuronal nets (Figure 10B). Our findings imply that the control of cell survival and targeting by the NT superfamily is an ancient mechanism of nervous system development. Further functions of the DNTs could also include synaptic and neuronal activity, learning, and memory. Our findings support the notion of a common origin for nervous system centralisation in evolution [122,123]. They suggest that in the course of evolution “elaborations of what went before” [124]—an available molecular mechanism involving the ancestral NTs—and “tinkering” [125] with NT signalling accompanied the diversification of nervous systems and behaviours.

The identification of DNTs bridges a void in neuronal studies using *Drosophila* as a model for understanding the brain. Conserved molecular mechanisms involving the NT superfamily may underlie aspects of retrograde transport, dendrite formation, axonal remodelling, synaptic plasticity, LTP, and learning and memory also in flies—all functions for which NTs are responsible in vertebrates. This work opens a wide range of opportunities to further the understanding of brain formation and evolution and to model human brain diseases using *Drosophila*.

## Materials and Methods

Details on methods can be found in Text S1. A summary is given below:

### Bioinformatics: Identification.

Full-length and Cystine-knot sequences from 28 known vertebrate NTs were used in PSI-BLAST searches (http://www.ncbi.nlm.nih.gov/blast/blast.cgi) against release 2 of the *Drosophila* genome. Carp (Cyprinus carpio) BDNF showed homology with *CG18318* both in BLAST and PSI-BLAST searches as the only hit in *Drosophila*. This hit was verified by reverse-BLAST. When DNT1 is used as a query in structure-based searches using FUGUE, it identifies with over 99% certainty the human neurotrophins, comprising BDNF, NGF, NT3, and NT4 as probable homologs.

### Structural alignment and model of the DNT1 protomer.

To verify the homology of DNT1 to NTs, we carried out structural alignments. The sequences for the NT Cystine-knot domains were aligned against the HOMSTRAD [126] (http://www-cryst.bioc.cam.ac.uk/homstrad/) entry of the nerve growth factor (NGF) family using FUGUE [44]. Using this alignment, a model of DNT1 was built with MODELLER [127].

### Phylogenetic analysis.

Phylogenetic analysis was attempted using sequences comprised with the Cysknot domain only, as sequences diverge considerably outside the Cysknot. Methods used were Maximum Parsimony, Neighbour Joining, and Maximum Likelihood.

### Identification of DNT1 and Spz insect orthologs.

The BLAST server at FlyBase (http://www.flybase.org/blast/) was used to identify orthologs of *DmNT1* and *DmSpz* in other insect species (see also: http://rana.lbl.gov/drosophila/).

### DNT1 cleavage prediction.

Cleavage prediction analysis using the ProP server (http://www.cbs.dtu.dk/services/ProP) reveals two high scores at positions 283 and 294. However, the sequence most likely to match the cleavage site of Spz by Easter is FSLSKKR RE at position 498.

### Search for DNT1 homologs in the cnidarian *Nematostella* and mollusc *Aplysia*

We searched for sequence homologs of DNT1 in the sequenced genome of Nematostella vectensis and the EST collections of N. vectensis and Aplysia californica.

### Genetics and homologous recombination.

For details of the mutants, alleles, transgenic lines of flies, and GAL4 driver lines of flies used, see Text S1. Null alleles for DNT1 were generated by homologous recombination using the ends-out protocol. The coding region of *DNT1*, including the ATG and the whole Cysknot domain, was replaced by the coding region of the *white* gene.

### Isolation of cDNA3, cloning, and transgenesis for gain-of-function and RNAi experiments.

The *DNT1* locus corresponds to *CG18318* from release 2 and *CG32244* plus *CG32242* from release 3 of the sequenced genome (http://www.flybase.org). Full-length cDNA3 was amplified by PCR from cDNA libraries. DNT1 (cDNA3) was sequenced and presents the following characteristics: MW 100, 315 kDa, PI: 6.17. DNT1 is 886 aa long, with a Signal Peptide (1–30 aa), a pro-domain (31–498 aa), a 102-aa Cysknot domain (499–601 aa), and an extended, disordered 285-aa COOH tail (602–886 aa). For further details on this and on the generation of gain-of-function and RNAi constructs for transgenesis, see Text S1.

### RT-PCR and Southern blots for verification of RNAi and homologous recombinants.

RT-PCR was used to verify that targeted RNAi in a heterozgygous mutant background resulted in a down-regulation of *DNT1* transcripts encoding the Cysknot. Under the same conditions, the null *DNT1^41^* mutants do not produce transcripts, whereas heterozygote embryos produce transcripts in normal levels.

### Cell culture transfections and western blotting.

Cell culture and western blotting were used to verify cleavage and dimerisation of DNT1.

### In situ hybridisations and immunohistochemistry.

These methods were carried out following standard protocols, except that for Toll stainings, embryos were fixed for 10 min.

### Microscopy.

Wide-field microscopy was carried out with Nomarski optics with a Zeiss Axioplan 2 and confocal microscopy with Leica SP2 and Radiance 2000 laser scanning confocal microscopes.

### DeadEasy software for the automatic quantification of apoptotic cells.

We purposely wrote DeadEasy software as an ImageJ plug-in, to quantify automatically cells stained with the apoptotic marker anti-cleaved Caspase-3 (M. G. Forero, J. A. Pennack, A. R. Learte, K. Kato, R. L. Griffiths, and A. Hidalgo, unpublished data). For details, see Text S1.

### Statistical analysis.

Statistical analyses of all experiments, with rationale, tests applied, confidence intervals, and *p*-values, are given in Text S1.

### Filming of adult locomotion.

Filming was carried out with a Motic camera mounted on a Leica MZ8 microscope and using Motic Images Plus 2.0 software.

### Accession numbers.

The *DNT1* cDNA sequences have been deposited in GenBank; for accession numbers, see text and Text S1.

## Supporting Information

Figure S1 Known Neurotrophins in Animal Evolution Diagrammatic evolutionary tree illustrating the NTs (red) in deuterostomes. NTs are missing and thought to have been lost in tunicates represented by *Ciona*. Trk receptors are present in molluscs, represented by *Aplysia*. No NT sequences had been found in protostomes prior to this work.
(909 KB TIF)

Figure S2 DNT1 Protein Sequence Relative to cDNA3, the protein sequences of the shorter cDNA1 and cDNA2 terminate at residue position 454 (arrow), which in cDNA1 and cDNA2 is followed immediately by a stop codon. p.c.s., predicted cleavage site.
(850 KB TIF)

Figure S3 High Sequence Divergence among Invertebrate NT Superfamily Members Phylogenetic trees using the Cysknot from all known NTs, representing the four vertebrate groups (*BDNF*, *NGF*, *NT3*, and *NT4*), the ancient *NT*s from lamprey (*LfNT*), *Amphioxus* (*BfNT*), sea urchin (*SpNT1*), and acorn worm (*SkNT*), *DNT1* orthologs in *Anopheles* (*AgNT1*) and D. pseudoobscura (*DpNT1*) and Spz (*Dm Spz*). Only the Cysknot was used, because there is considerable sequence divergence outside the Cysknot. The structural alignment shown in Figure 1B was used. The trees were built using three methods: (A, B, and C) Maximum Parsimony; (D) Neighbour Joining; (E) Maximum Likelihood. Numbers indicate percent bootstrap with 1,000 bootstraps in all trees.
(A) This tree is unrooted and shows that sequence similarity is higher within the two clades of vertebrate *NT*s and insect sequences, and that the insect sequences are closer to the ancient *NT*s represented by *SkNT*, *SpNT*, and *BfNT.*
(B–E) These trees are rooted with the only two available alternative roots: TGFβ from the pufferfish (*Fugu*) and coagulogen from the horseshoe crab. TGFβ belongs to the Cysknot superfamily (which also includes PDGF), but the TGFβ Cysknot is different in structure form the NT Cysknot. *Fugu* is an ancient fish, which is more useful than using a more evolved sequence. Coagulogen from horseshoe crab was used because it has a Cysknot resembling Spz, and horseshoe crabs are very primitive. There are no more ancient NT superfamily Cysknot sequences that we could have used to root the trees. The coagulogen sequence was added to the alignment in Figure 1B based on the structure-based alignment in reference [1]. In all the trees, insect *DNT1* and *spz* form a separate clade from deuterostome *NT*s, which is supported by the high conservation of these genes within insects. (B and C) With Maximum Parsimony, rooting the trees either with TGFβ or coagulogen reveals closer similarity of insect sequences to the invertebrate deuterostome *NT*s *SkNT*, *SpNT*, and *BfNT*. The tree in (B) lacks acorn worm *SkNT* sequence. (D and E) Within this low margin of sequence identity (<30%), coagulogen is not sufficiently different from the NT Cysknot. Interestingly, once again, acorn worm *NT SkNT* appears to be the most diverged of deuterostome *NT*s. To conclude, *DmNT1* and *spz* as well as the ancient *NT*s (*BfNT*, *SpNT*, and *SkNT*) have diverged considerably in sequence, precluding the phylogeny to resolve: (1) The trees do not resolve the relationship between *DmNT1*, *spz*, and the vertebrate *NT*s. (2) The relationships of the ancient *NT*s to the vertebrate *NT*s and the insect clades varies with the trees, particularly in the case of acorn worm (*SkNT*). Structural alignment had also revealed a closer similarity of *SkNT* to *DNT1* and *spz* as well as the vertebrate *NT*s than *BfNT* or *SpNT*. (3) Although Maximum Likelihood is the best method for distantly related sequences, the low bootstrap values in (E) indicate that sequence divergence is too high to resolve the phylogeny.
(1.27 MBTIF).

Figure S4 DeadEasy Software for the Automatic Quantification of Apoptosis In Vivo (A) Anti-cleaved Caspase-3 (Caspase-3) is a reliable apoptotic marker. Codetection of the apoptotic markers TUNEL (magenta) and Caspase (green) in a single 0.5-μm section of a stained embryonic VNC. Single-channel higher magnification details of one cell are shown on the right.
(B) How DeadEasy software quantifies cells. We wrote DeadEasy as an Image-J plug-in. Whole embryos are stained in vivo with Caspase-3 and the whole thickness of the ventral nerve cord (VNC) is scanned under the confocal microscope, sections are 0.25 μm apart, over 100 sections per VNC. A region of interest (ROI) is drawn over the lateral edges of the VNC to eliminate epidermal apoptosis from the counts. DeadEasy is run as an Image-J plug-in throughout the whole stack. Each individual section is processed to identify objects. Identified cells are labelled throughout the stack, and they are classified in 3-D according to minimum volume and also based on minimum pixel intensity. DeadEasy produces a message with the total number of Caspase-3 cells counted in about 1 min per embryonic VNC (or stack). For details see Text S1.
(3.67 MB TIF)

Figure S5 *spz* and *DNT2* Orthologs in Insect Species Alignment of the Cysknot domain of (A) *spz* and (B) *DNT2* to their orthologs from insects with sequenced genomes, including 12 *Drosophila* species, three mosquito species (Anopheles aegypti, A. gambiae, and Culex pipiens), beetle (Tribolium castaneum), silk moth (Bombyx mori), and human body louse (*Pediculus humanus corporis*). Identical residues are shown in white over red; conservative substitutions in red. There is conservation of *spz* and *DNT2* in insects within the Cysknot, lower for *spz*. For accession numbers see Text S1.
(2.06 MB TIF)

Figure S6 Muscles Develop Normally in *DNT1* and *DNT2* Mutants (A) Anti-Myosin stage 17 stained embryos, three different focal planes are shown from top to bottom. Arrows point at muscles shown in each focal plane and which coincide with the expression domains of *DNT1*, *DNT2*, and *spz*. No muscles defects were observed in stage 17 stained embryos. Some stage 13–16 *spz^2^* and *DNT2^e03444^* mutant embryos have abnormal morphology and CNS defects, and the penetrance of these abnormal embryos can increase to 20%–40% in the double- and triple-mutant embryos. These severe phenotypes might be a consequence of earlier developmental defects in dorsoventral patterning, as they can be seen prior to muscle development. To ensure that only zygotic functions are analysed, we focus on stage 17 embryos.
(B) Targeting defects occur independently of muscle defects: here, three different focal planes are shown to indicate normal muscle patterning with loss of axonal targeting. Arrowheads indicate muscles, arrows axons. There are occasional muscle defects at stage 17, particularly in triple-mutant embryos. Thus, it is possible that DNTs may also play functions in the muscle. Axon guidance and targeting phenotypes can be dissociated from muscle phenotypes.
(13.48 MB TIF)

Table S1 Features of Invertebrate Deuterostomian Neurotrophins (1.13 MB TIF)

Table S2 A NT Superfamily Cysknot Is Present in DNT1/Spz2, Spz, and DNT2/Spz5 (48 KB DOC)

Table S3 Synergistic Interactions between Mutations in *DNT1*, *DNT2*, and *spz*
(28 KB DOC)

Table S4 Locomotion Deficits: Video Details (26 KB DOC)

Text S1 Detailed Methods (116 KB DOC)

Video S1 Wild-Type Adult Fly (182 KB MPG)

Video S2 *DNT1^41^ DNT2^e03444^* Double-Mutant Adult Fly Fails to Estimate Location of Rim (1.23 MB MPG)

Video S3 *DNT1^41^ DNT2^e03444^* Double-Mutant Adult Fly Falls off Rim (MB MPG).

Video S4 *DNT1^41^ DNT2^e03444^* Double-Mutant Adult Fly Is Sluggish (496 KB MPG)

Video S5 *DNT1^41^ DNT2^e03444^* Double-Mutant Adult Fly Wobbles (3.38 MB MPG)

Video S6 *DNT1^41^ DNT2^e03444^* /*DNT1^41^ Df(3L)6092* Double-Mutant Adult Fly Fails to Estimate Rim (1.30 MB MPG)

Video S7 *DNT1^41^ DNT2^e03444^* / *DNT1^41^ Df(3L)6092* Double-Mutant Adult Fly Is Slow (1.22 MB MPG)

Video S8 *DNT1^41^ DNT2^e03444^* / *DNT1^41^ Df(3L)6092* Double-Mutant Adult Fly Wobbles (3.31 MB MPG)

Video S9 *spz^2^/spz^2^* Adult Mutant Fly Is Extremely Uncoordinated (1.71 MB MPG)

## Abbreviations

aa: amino acid
BDNF: brain-derived neurotrophic factor
Caspase-3: anti-cleaved-Caspase-3
Cysknot: cystine-knot
CNS: central nervous system
DNT1: *Drosophila* neurotrophin 1
EST: expressed sequence tag
ISN: intersegmental nerve
LTP: long-term potentiation
NGF: nerve growth factor
NOCD: naturally occurring cell death
NT: neurotrophin
PNS: peripheral nervous system
RNAi: RNA interference
RT-PCR: reverse transcriptase polymerase chain reaction
SN: segmental nerve
Spz: Spätzle
TNF: tumour necrosis factor
VNC: ventral nerve cord
