# Supplementary figures and images for: Drosophila Neurotrophins Reveal a Common Mechanism for Nervous System Formation
Source: PLoS Biol. 2008 Nov 18;6(11):e284. doi: 10.1371/journal.pbio.0060284 (PMC2586362; doi:10.1371/journal.pbio.0060284)

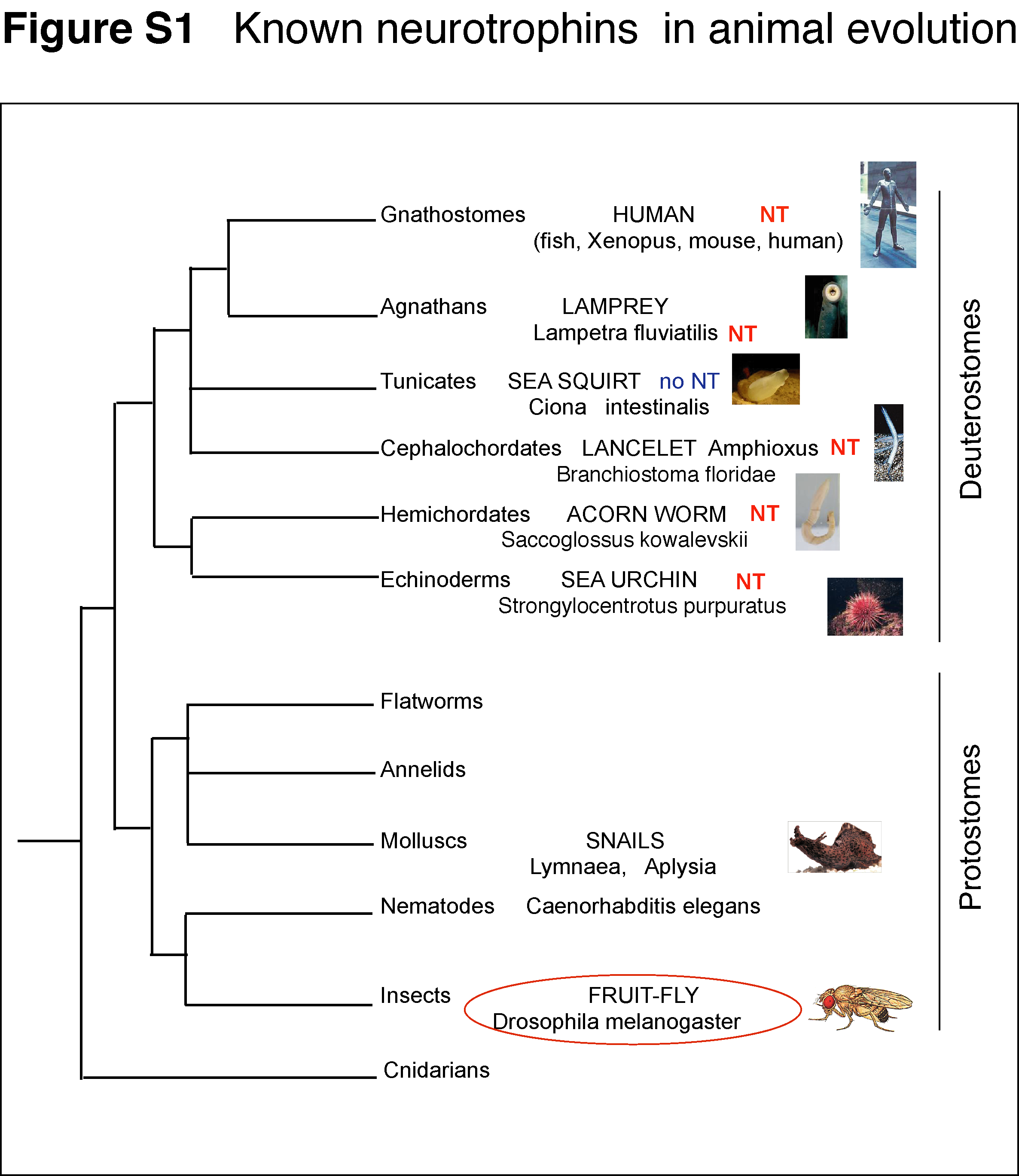

Supplement: Figure S1 — Diagrammatic evolutionary tree illustrating the NTs (red) in deuterostomes. NTs are missing and thought to have been lost in tunicates represented by Ciona. Trk receptors are present in molluscs, represented by Aplysia. No NT sequences had been found in protostomes prior to this work. (909 KB TIF) [file pbio.0060284.sg001.tif]

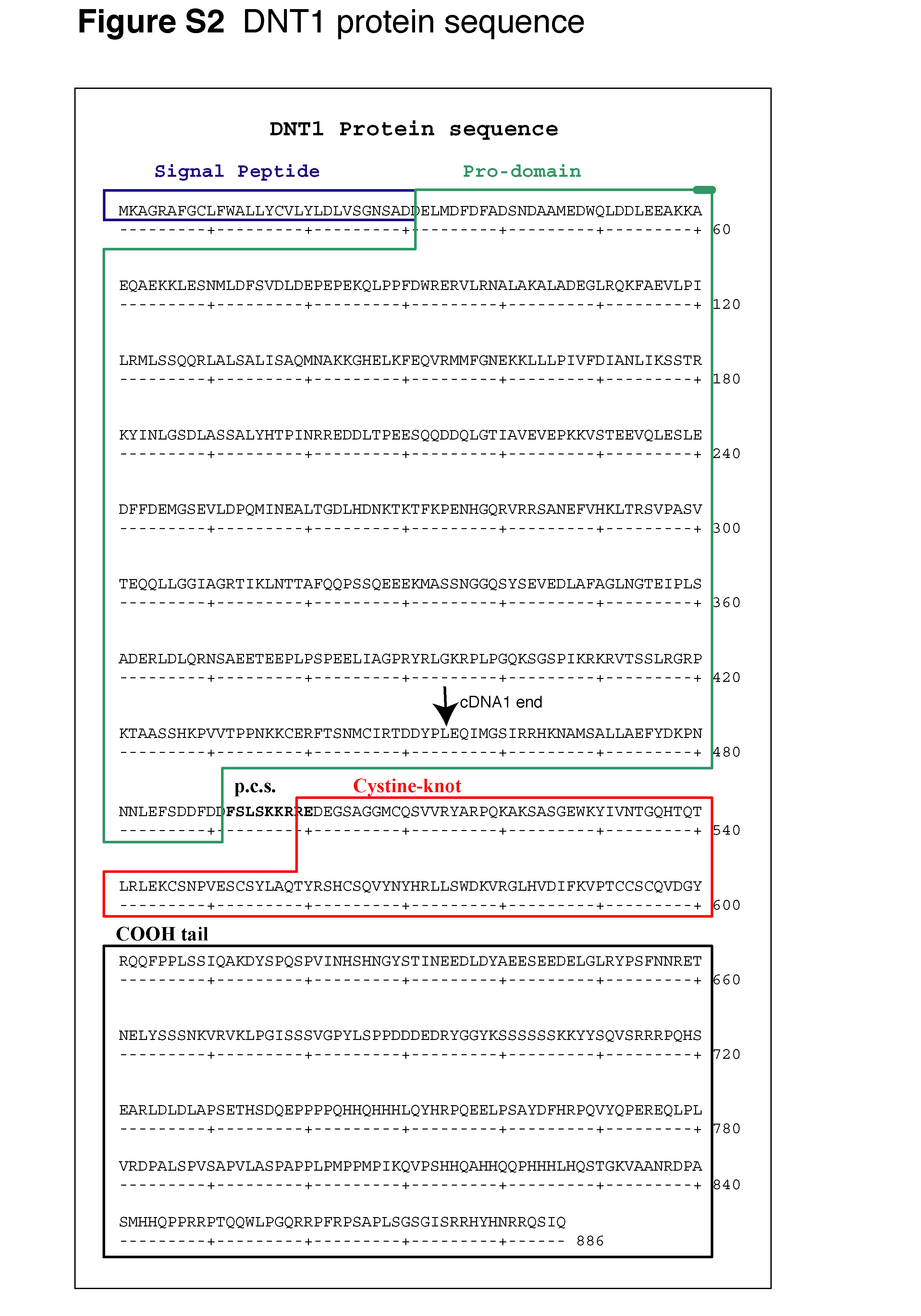

Supplement: Figure S2 — Relative to cDNA3, the protein sequences of the shorter cDNA1 and cDNA2 terminate at residue position 454 (arrow), which in cDNA1 and cDNA2 is followed immediately by a stop codon. p.c.s., predicted cleavage site. (850 KB TIF) [file pbio.0060284.sg002.tif]

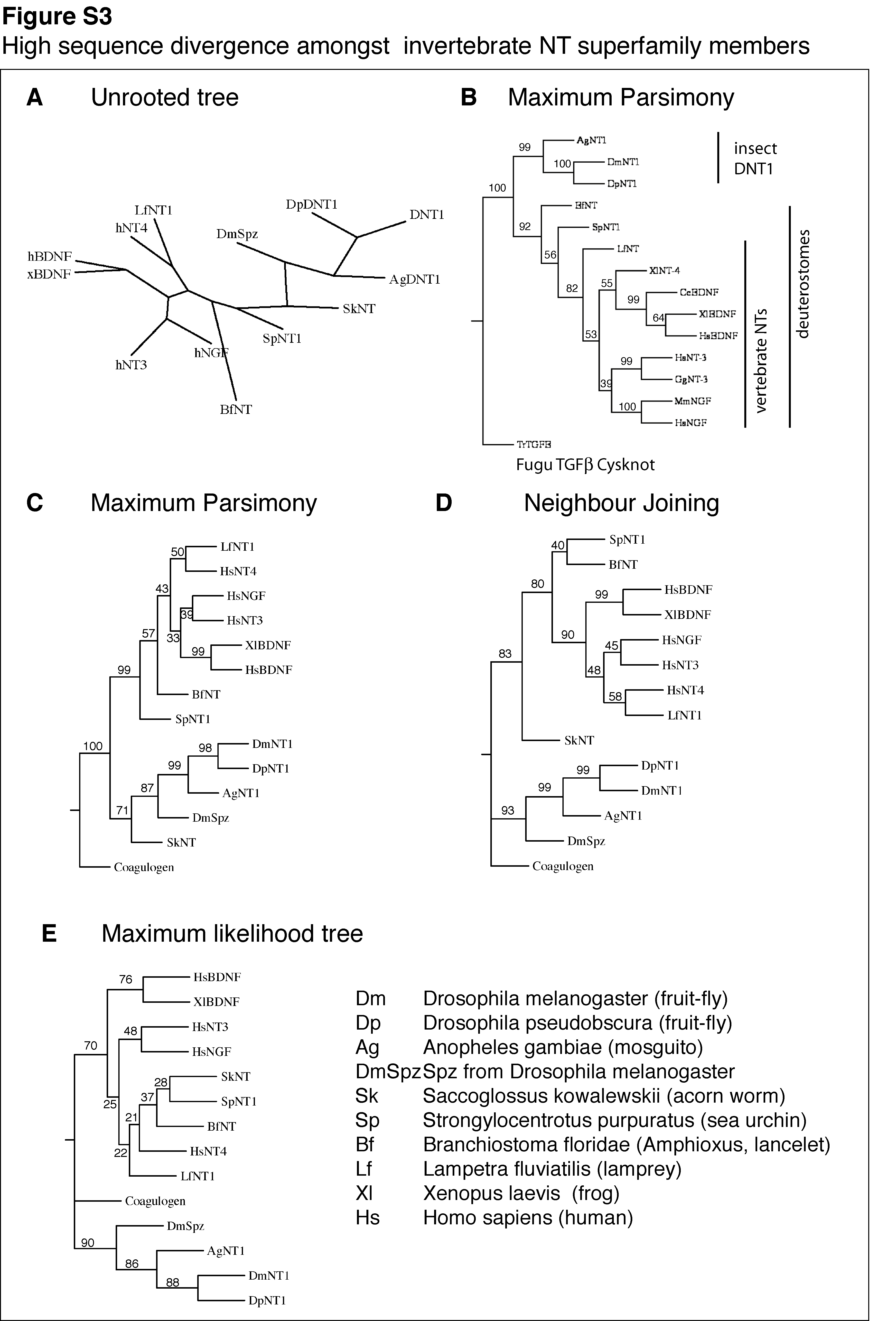

Supplement: Figure S3 — Phylogenetic trees using the Cysknot from all known NTs, representing the four vertebrate groups (BDNF, NGF, NT3, and NT4), the ancient NTs from lamprey (LfNT), Amphioxus (BfNT), sea urchin (SpNT1), and acorn worm (SkNT), DNT1 orthologs in Anopheles (AgNT1) and D. pseudoobscura (DpNT1) and Spz (Dm Spz). Only the Cysknot was used, because there is considerable sequence divergence outside the Cysknot. The structural alignment shown in Figure 1B was used. The trees were built using three methods: (A, B, and C) Maximum Parsimony; (D) Neighbour Joining; (E) Maximum Likelihood. Numbers indicate percent bootstrap with 1,000 bootstraps in all trees. (A) This tree is unrooted and shows that sequence similarity is higher within the two clades of vertebrate NTs and insect sequences, and that the insect sequences are closer to the ancient NTs represented by SkNT, SpNT, and BfNT. (B–E) These trees are rooted with the only two available alternative roots: TGFβ from the pufferfish (Fugu) and coagulogen from the horseshoe crab. TGFβ belongs to the Cysknot superfamily (which also includes PDGF), but the TGFβ Cysknot is different in structure form the NT Cysknot. Fugu is an ancient fish, which is more useful than using a more evolved sequence. Coagulogen from horseshoe crab was used because it has a Cysknot resembling Spz, and horseshoe crabs are very primitive. There are no more ancient NT superfamily Cysknot sequences that we could have used to root the trees. The coagulogen sequence was added to the alignment in Figure 1B based on the structure-based alignment in reference [1]. In all the trees, insect DNT1 and spz form a separate clade from deuterostome NTs, which is supported by the high conservation of these genes within insects. (B and C) With Maximum Parsimony, rooting the trees either with TGFβ or coagulogen reveals closer similarity of insect sequences to the invertebrate deuterostome NTs SkNT, SpNT, and BfNT. The tree in (B) lacks acorn worm SkNT sequence. (D and E) Within [file pbio.0060284.sg003.tif]

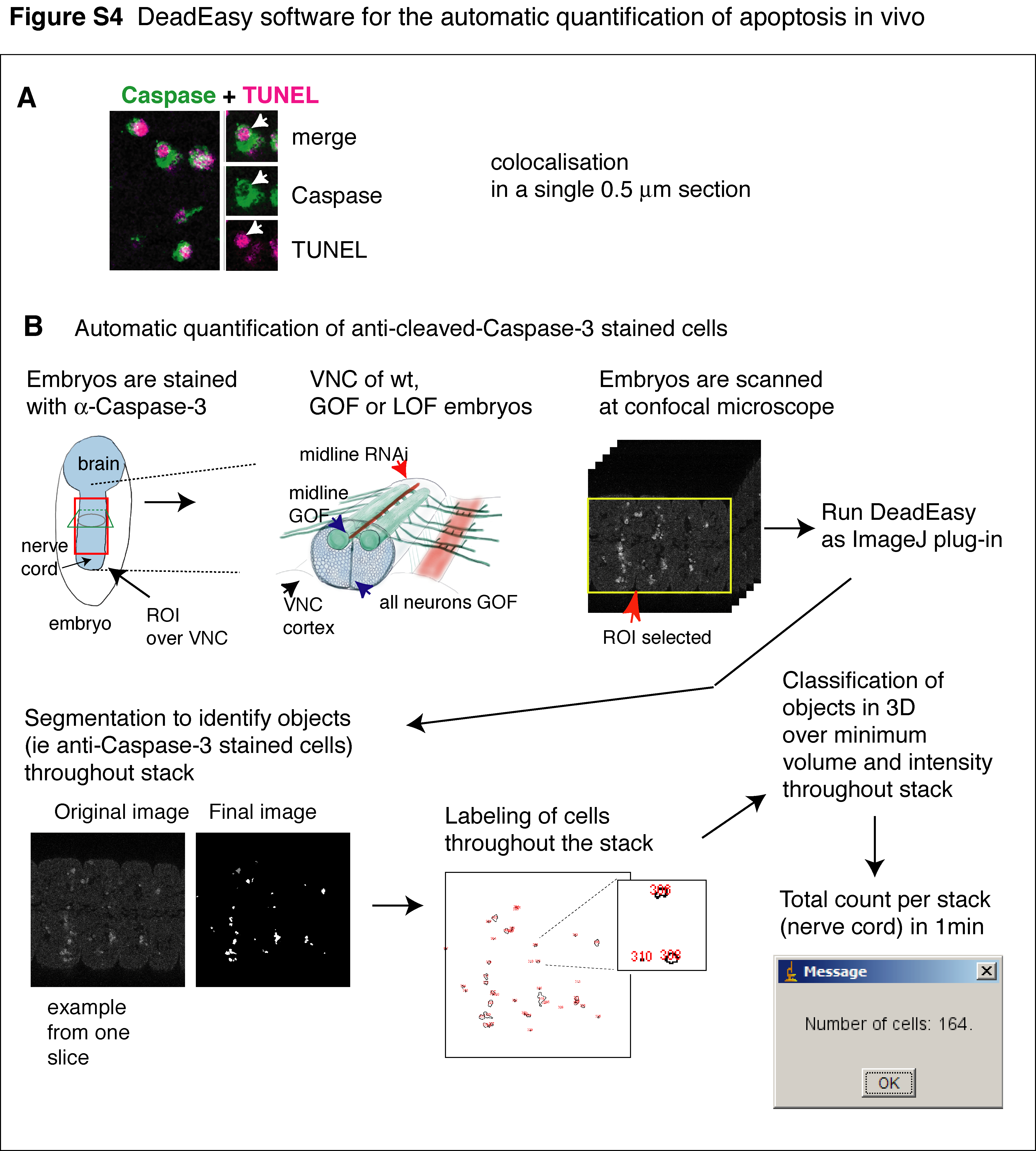

Supplement: Figure S4 — (A) Anti-cleaved Caspase-3 (Caspase-3) is a reliable apoptotic marker. Codetection of the apoptotic markers TUNEL (magenta) and Caspase (green) in a single 0.5-μm section of a stained embryonic VNC. Single-channel higher magnification details of one cell are shown on the right. (B) How DeadEasy software quantifies cells. We wrote DeadEasy as an Image-J plug-in. Whole embryos are stained in vivo with Caspase-3 and the whole thickness of the ventral nerve cord (VNC) is scanned under the confocal microscope, sections are 0.25 μm apart, over 100 sections per VNC. A region of interest (ROI) is drawn over the lateral edges of the VNC to eliminate epidermal apoptosis from the counts. DeadEasy is run as an Image-J plug-in throughout the whole stack. Each individual section is processed to identify objects. Identified cells are labelled throughout the stack, and they are classified in 3-D according to minimum volume and also based on minimum pixel intensity. DeadEasy produces a message with the total number of Caspase-3 cells counted in about 1 min per embryonic VNC (or stack). For details see Text S1. (3.67 MB TIF) [file pbio.0060284.sg004.tif]

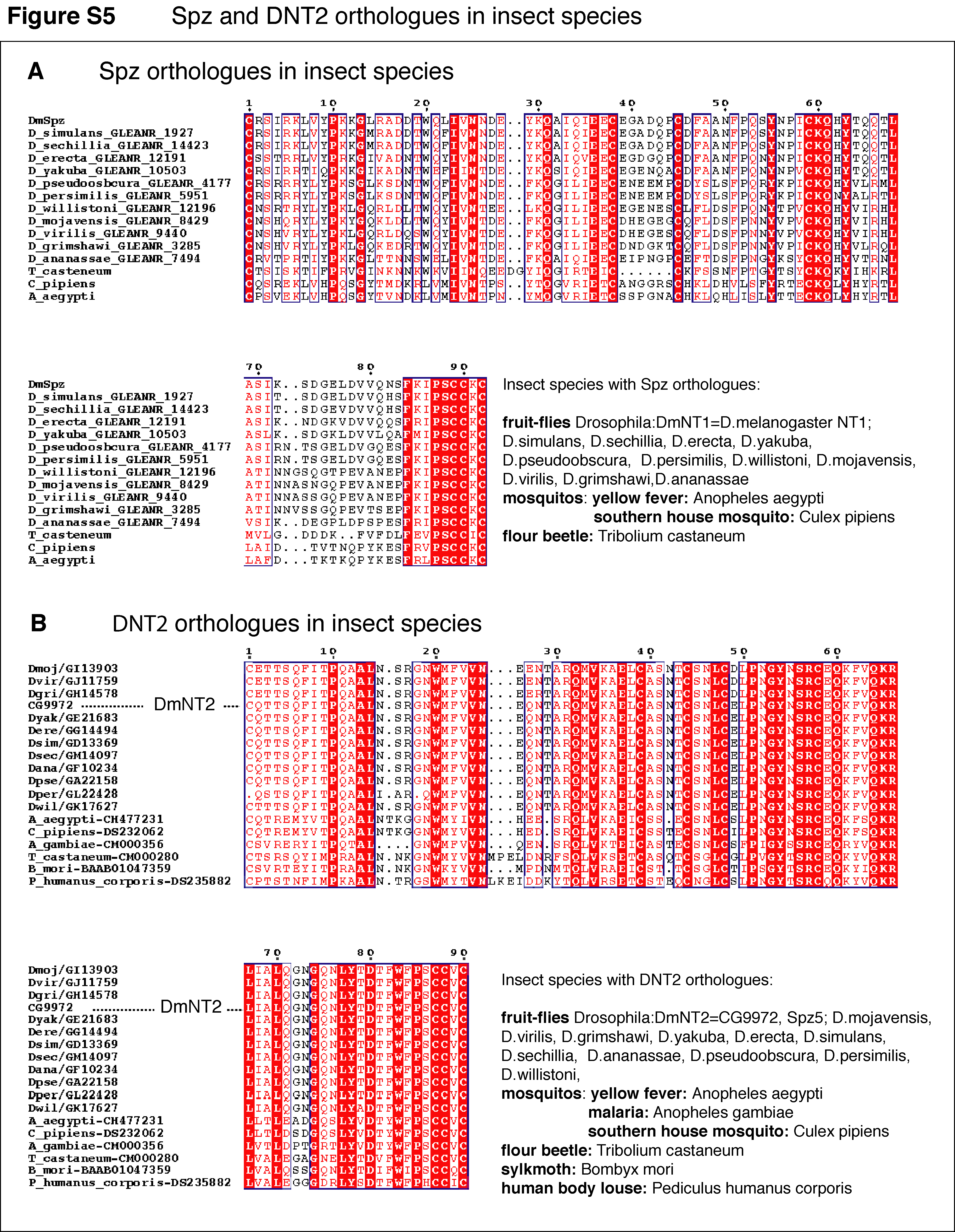

Supplement: Figure S5 — Alignment of the Cysknot domain of (A) spz and (B) DNT2 to their orthologs from insects with sequenced genomes, including 12 Drosophila species, three mosquito species (Anopheles aegypti, A. gambiae, and Culex pipiens), beetle (Tribolium castaneum), silk moth (Bombyx mori), and human body louse (Pediculus humanus corporis). Identical residues are shown in white over red; conservative substitutions in red. There is conservation of spz and DNT2 in insects within the Cysknot, lower for spz. For accession numbers see Text S1. (2.06 MB TIF) [file pbio.0060284.sg005.tif]

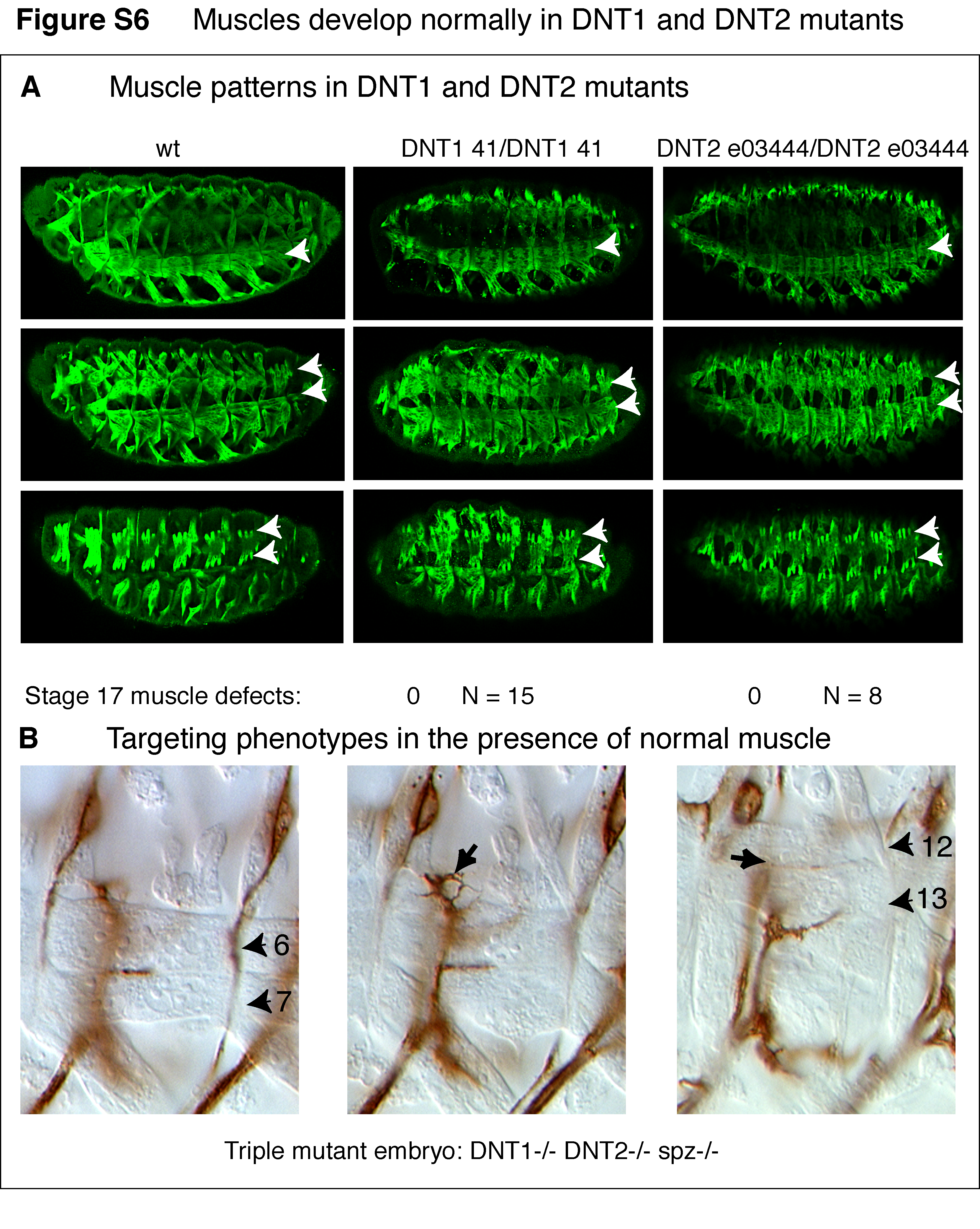

Supplement: Figure S6 — (A) Anti-Myosin stage 17 stained embryos, three different focal planes are shown from top to bottom. Arrows point at muscles shown in each focal plane and which coincide with the expression domains of DNT1, DNT2, and spz. No muscles defects were observed in stage 17 stained embryos. Some stage 13–16 spz2 and DNT2e03444 mutant embryos have abnormal morphology and CNS defects, and the penetrance of these abnormal embryos can increase to 20%–40% in the double- and triple-mutant embryos. These severe phenotypes might be a consequence of earlier developmental defects in dorsoventral patterning, as they can be seen prior to muscle development. To ensure that only zygotic functions are analysed, we focus on stage 17 embryos. (B) Targeting defects occur independently of muscle defects: here, three different focal planes are shown to indicate normal muscle patterning with loss of axonal targeting. Arrowheads indicate muscles, arrows axons. There are occasional muscle defects at stage 17, particularly in triple-mutant embryos. Thus, it is possible that DNTs may also play functions in the muscle. Axon guidance and targeting phenotypes can be dissociated from muscle phenotypes. (13.48 MB TIF) [file pbio.0060284.sg006.tif]

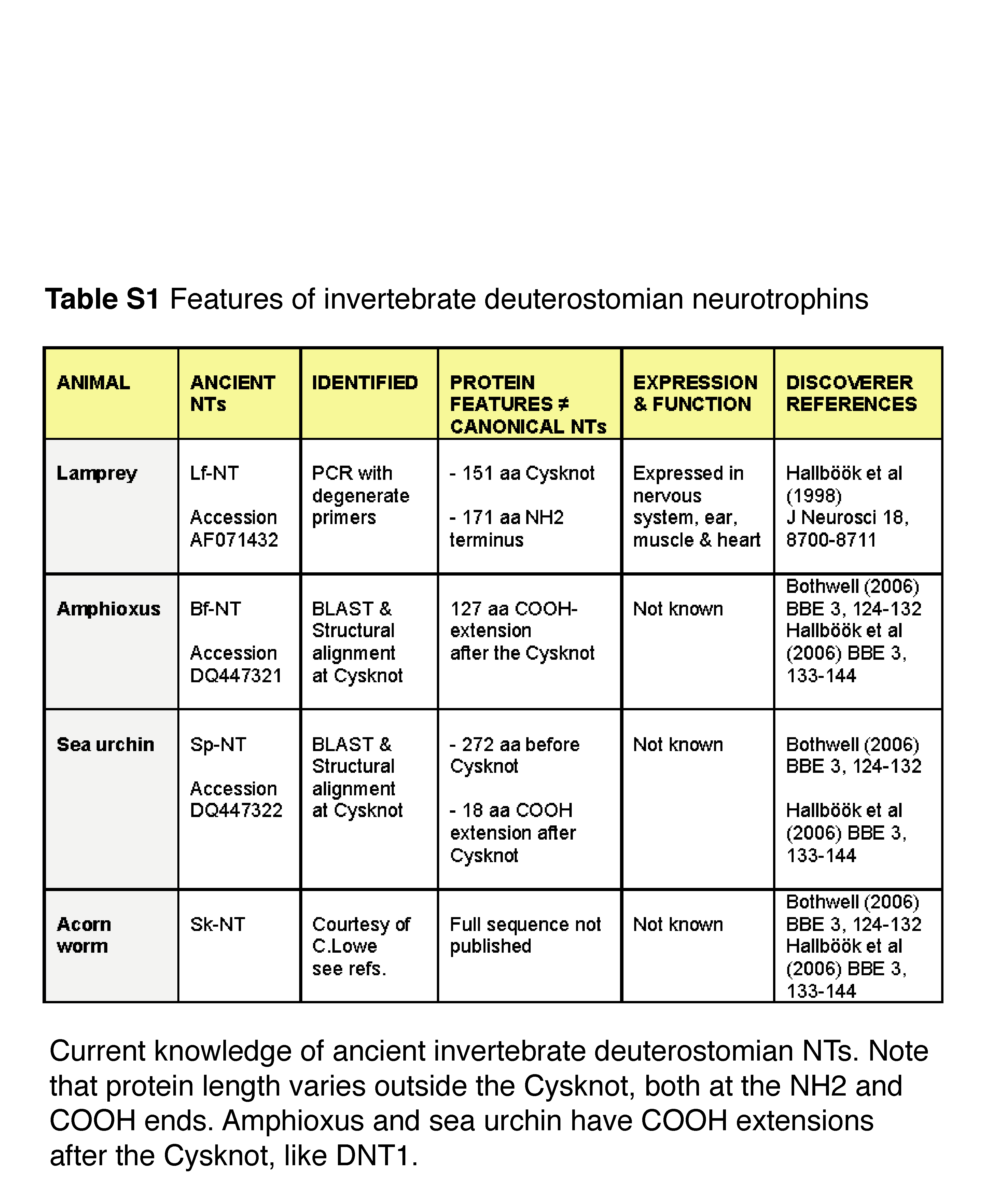

Supplement: Table S1 — (1.13 MB TIF) [file pbio.0060284.st001.tif]
