# Supplementary material for: Drosophila Neurotrophins Reveal a Common Mechanism for Nervous System Formation
Source: PLoS Biol. 2008 Nov 18;6(11):e284. doi: 10.1371/journal.pbio.0060284 (PMC2586362; doi:10.1371/journal.pbio.0060284)
Supplement: Table S2 — (48 KB DOC) [file pbio.0060284.st002.doc]

# Table S2 A NT superfamily Cysknot is present in DNT1/Spz2, Spz and DNT2/Spz5

**Abbreviations**: CI-CVI: Cysteines I to VI within the Cysknot. CI-CVI =NTs: canonical vertebrate NT Cysknot with conserved Cysteines. +1 = Sk-NT: extra Cysteine like in acorn worm NT (see below §); ≠ from all NTs: these Cysknots differ from the canonical NT Cysknot. *** FUGUE scores:** the threshold value is 6, above which structural similarity to a protein of known structure (i.e. inferred from crystals) is with 99% confidence. Fugue score = 6 reveals similarity with 95% confidence. Scores below 6 indicate no significant structural homology. Thus FUGUE revelas a closer similarity of DNT1 to NTs than to Coagulogen. Previous analysis by Mizuguchi et al (1998) and Parker et al (2001) did not employ FUGUE.

**§ Cysknot features:** DNT1, Spz, Spz5 and Sk-NT (as well as Spz3 and Spz4) have, as well as the canonical 6 Cysteines from vertebrate NTs, a seventh Cysteine prior to the canonical 5^th^ Cys. This 7^th^ Cys is thought to stabilise the Cysknot dimers. Spz5 has an extra Cys. The number and distribution of Cysteines in Spz3, 4 and 6 differ from the canonical NT Cysknot. The Cysknot in Spz6 is very different form the canonical NT Cysknot.

| **Spz paralogue** | **FUGUE score***  **vs h-NTs**  **>6= 99%C** | **Conserved**  **Cysteines**  **as in NT Cysknot §** | **Cysknot SIZE**  **NT=106 aa** | **% Identity** | **EXPRESSION**  **& Function** | **REFERENCES** |
| --- | --- | --- | --- | --- | --- | --- |
| **DNT1/Spz2** | 7.38 vs  NTs  3.77 vs Coagulogen | **CI-CVI = NTs**  **+1C before CV = Sk-NT** | 102 aa | 26 % to  BDNF | Expressed at all stages;  NT function | This work;  Parker et al (2001) Proteins 45, 71-80 |
| **Spz** | **7.16** | **CI-CVI = NTs**  **+1C = Sk-NT** | 106 aa | 19% to  NGF | Expressed at least in embryo;  NT function | This work;  Parker et al (2001) Proteins;  DeLotto&DeLotto (1998) MOD 72;  Mizuguchi et al (1998) TIBS 23 |
| **Spz3** | 5.4 | CI-CVI = NTs  +2 C  ≠ from all NTs | 100 aa | 51% to Spz4 | Not known;  possibly like spz4? | This work;  Parker et al (2001) Proteins  45, 71-80 |
| **Spz4** | 6.79 | CI-CVI = NTs  +2 C  ≠ from all NTs | 97 aa  lacks conserved intron | 29% to Coagulogen  51% to Spz3 | Larva, adult.  Expression  Increases upon immune challenge | This work;  Parker et al (2001) Proteins 45, 71-80 |
| **DNT2/Spz5** | **7.63** | **CI-CVI = NTs**  **+1C = Sk-NT**  **+1C** | 102 aa | 26% to Spz in Cysknot | Expressed at least in embryo;  NT function | This work;  Parker et al (2001) Proteins 45, 71-80 |
| **Spz6** | 2.49 | No CIV  No +1C  + 3C extra  at different positions  ≠ from all NTs | 152 aa  85 aa between CIII and CV differ  lacks conserved intron | diverged | Expressed in embryo, larvae, CNS, PNS | This work;  Parker et al (2001) Proteins 45, 71-80 |
