# Supplementary material for: Drosophila Neurotrophins Reveal a Common Mechanism for Nervous System Formation
Source: PLoS Biol. 2008 Nov 18;6(11):e284. doi: 10.1371/journal.pbio.0060284 (PMC2586362; doi:10.1371/journal.pbio.0060284)
Supplement: Table S3 — (28 KB DOC) [file pbio.0060284.st003.doc]

TABLES S3

Synergistic interactions between mutations in *DNT1, DNT2* and *spz*

| **GENE & ALLELE** | **MOLECULAR**  **LESION** | **GENOTYPE** | **LETHALITY** | LOCOMOTION **DEFICITS**  **See films** |
| --- | --- | --- | --- | --- |
| **DNT1** | null | DNT1^41^/DNT1^41^ | Viable & fertile | Not apparent. |
| **DNT1** | null | DNT1^41^/TM6B | Viability of DNT1-/- compromised. |  |
| **DNT2** | PBac insertion into intron | DNT2 ^e03444^/  DNT2 ^e03444^ | Viable and fertile | Not apparent |
| **spz** | EMS induced mutation in pro-domain | spz^2^/TM6B | Homozygous **semi-lethal**  7.12% escapers (n= 407) | **Homozygous mutant flies have severe locomotion deficit.**  **100% penetrance** |
| **spz** | EMS-induced mutation in Cysknot domain | spz^3^/TM6B | Homozygous lethal | N/A |
| **DNT1 DNT2** | Double mutant | DNT1^41^ DNT2 ^e03444^ /TM6B | Homozygous lethal at 18°C  Is it due to competition? | N/A |
| **DNT1 DNT2** | Double mutant | DNT1^41^ DNT2^e03444^/ DNT1^41^ DNT2 ^e03444^ | Homozygous viable also at 18°C, some larval lethality | **Locomotion deficits**  **Severe: 41% n=17**  **Mild: 11.7%** |
| **DNT1 DNT2** | Double mutant | DNT1^41^ Df6092 /TM6B | Homozygous lethal | N/A |
| **DNT1 DNT2** | Double mutant | DNT1^41^ DNT2 ^e03444^ /DNT1^41^ Df6092 | **Some larval lethality** | **Locomotion deficits**  **Severe: 3.3% n=30**  **Mild: 10%** |
| **DNT1 spz** | Double mutant | DNT1^41^ spz^2^ / DNT1^41^ spz^2^ | **Homozygous lethal** | N/A |
| **DNT1 DNT2 spz** | Triple mutant | DNT1^41^ DNT2 ^e03444^ spz^2^/  DNT1^41^ DNT2 ^e03444^ spz^2^ | **Homozygous lethal** | N/A |
| **DNT1 DNT2 spz/**  **DNT1 DNT2** | Triple mutant in trans over double | DNT1^41^ DNT2 ^e03444^ spz^2^/  DNT1^41^ DNT2 ^e03444^ | ? | ? |
