# Supplementary material for: Drosophila Neurotrophins Reveal a Common Mechanism for Nervous System Formation
Source: PLoS Biol. 2008 Nov 18;6(11):e284. doi: 10.1371/journal.pbio.0060284 (PMC2586362; doi:10.1371/journal.pbio.0060284)
Supplement: Table S4 — (26 KB DOC) [file pbio.0060284.st004.doc]

**Table S4 Locomotion deficits: film details.**

| **FILM** | **GENOTYPE** | **FLY** | **PHENOTYPE** | APPROXIMATE PENETRANCE |
| --- | --- | --- | --- | --- |
| 1 Wild-type | Oregon-R | 6c | Fly placed on centre of the Petri dish gets quickly on rim and runs very fast along it, round and round, doesn’t fall over. | 100%  N = 10 flies |
| **2 DNT1-DNT2-** | Homozygous DNT2^e03444^DNT1^41^ | 1 of 9a | Severe. Slow, uncoordinated movements. Reaches for something as if not aware of the edges of the rim. | Failing to estimate rim: 5.8%  N =17 |
| **3 DNT1-DNT2** | Homozygous DNT2^e03444^DNT1^41^ | 1 of 9c  same fly as 1 of 9a | Severe. As above, and failing to estimate the edges of the rim, it falls off | Falling off: 23.5%  N =17 |
| **4 DNT1-DNT2-** | Homozygous DNT2^e03444^DNT1^41^ | 4b | Severe. Extremely sluggish, after a while it walks. | Slow to very sluggish:  35%  N =17 |
| **5 DNT1-DNT2-** | Homozygous DNT2^e03444^DNT1^41^ | 8a | Mild. Wobbles walking along rim, it falls off. | Wobbling: 11.7%  N =17 |
| **6 DNT1-DNT2-** | DNT2^e03444^DNT1^41^/ Df6092 DNT1^41^ | 5a | Severe. Slow, uncoordinated movements. Reaches for something as if not aware of the edges of the rim. | Failing to estimate rim:  3.3%  N=30 |
| **7 DNT1-DNT2-** | DNT2^e03444^DNT1^41^/ Df6092 DNT1^41^ | 5c  same fly as 5a | Severe. Slow, uncoordinated movements. | Slow:  3.3%  N = 30 |
| **8 DNT1-DNT2-** | DNT2^e03444^DNT1^41^/ Df6092 DNT1^41^ | 17a | Mild. Wobbles walking along rim. | Wobbling: 10%  N =30 |
| **9 Spz-** | Homozygous spz^2^ | 9  movie 2 | Extremely uncoordinated, difficulties walking, when toppled over cannot right itself.  In other flies, it can be more severe than this. | 100%  n = 9 flies |

The penetrance of DNT1^41^DNT2^e03444^ locomotion phenotypes is likely lower as not all phenotypically normal flies were filmed.

Please refer to attached films.
