# Supplementary material for: Drosophila Neurotrophins Reveal a Common Mechanism for Nervous System Formation
Source: PLoS Biol. 2008 Nov 18;6(11):e284. doi: 10.1371/journal.pbio.0060284 (PMC2586362; doi:10.1371/journal.pbio.0060284)
Supplement: Text S1 — (116 KB DOC) [file pbio.0060284.sd001.doc]

# SUPPORTING INFORMATION

# Drosophila neurotrophins reveal a common mechanism for nervous system formation

B. Zhu^1^*, J. A.Pennack^1^*, P. McQuilton^1,2^, M. G. Forero^1^, K. Mizuguchi^3,4,5^, B. Sutcliffe^1^, C. Gu^1^, J. C.Fenton^1^ and A. Hidalgo^1^‡

Table S1 to Table S4

Figure S1 to Figure S6

Films S1 to S9

Supporting Figure legends

Text S1: Supporting Materials and Methods

Supporting Materials and Methods References

FIGURE LEGENDS

Figure S1 Known NTs in animal evolution. Diagrammatic evolutionary tree illustrating the NTs (red) in deuterostomes. NTs are missing and thought to have been lost in tunicates represented by Ciona. Trk receptors are present in mollusks, represented by Aplysia. No NT sequences had been found in protostomes prior to this work.

# Figure S2 DNT1protein sequence. p.c.s.: predicted cleavage site. Relative to cDNA3, the protein sequences of the shorter cDNA1 and cDNA2 terminate at residue position 454 (arrow), which in cDNA1 and cDNA2 is followed immediately by a stop codon.

# Figure S3 High sequence divergence amongst invertebrate NT superfamily members. Phylogenetic trees using the Cysknot from all known NTs, representing the four vertebrate groups (BDNF, NGF, NT3 and NT4), the ancient NTs from lamprey (LfNT), Amphioxus (BfNT), sea urchin (SpNT1) and acorn worm (SkNT), DNT1 orthologues in Anopheles (AgNT1) and D.pseudoobscura (DpNT1) and Spz (Dm Spz). Only the Cysknot was used because there is considerable sequence divergence outside the Cysknot. The structural alignment shown in Fig.1b was used. The trees were built using three methods: (A,B,C) Maximum Parsimony; (D) Neighbour Joining; (E) Maximum Likelihood. Numbers indicate percent bootstrap with 1000 bootstraps in all trees. (A) This tree is un-rooted and shows that sequence similarity is higher within the two clades of vertebrate NTs and insect sequences, and that the insect sequences are closer to the ancient NTs represented by SkNT, SpNT and BfNT. (B-E) These trees are rooted with the only two available alternative roots: TGFβ from the pufferfish (Fugu) and Coagulogen from the horseshoe crab. TGFβ belongs to the Cysknot superfamily (which also includes PDGF), but the TGFβ Cysknot is different in structure form the NT Cysknot. Fugu is an ancient fish, which is more useful than using a more evolved sequence. Coagulogen from horseshoe crab was used because it has a Cysknot resembling Spz and horseshoe crabs are very primitive. There are no more ancient NT superfamily Cysknot sequences that we could have used to root the trees. The Coagulogen sequence was added to the alignment in Fig.1B based on the structure-based alignment in reference[1]. In all the trees, insect DNT1 and Spz form a separate clade from deuterostome NTs, which is supported by the high conservation of these genes within insects. (B,C) With Maximum Parsimony, rooting the trees either with TGFβ or Coagulogen reveals closer similarity of insect sequences to the invertebrate deuterostome NTs SkNT, SpNT and BfNT. The tree in (B) lacks acorn worm SkNT sequence. (D,E) Within this low margin of sequence identity (<30%) Coagulogen is not sufficiently different from the NT Cysknot. Interestingly, once again acorn worm NT SkNT appears to be the most diverged of dueterostome NTs. To conclude, DmNT1 and Spz as well as the ancient NTs (BfNT, SpNT and SkNT) have diverged considerably in sequence precluding the phylogeny to resolve: 1, The trees do not resolve the relationship between DmNT1, Spz and the vertebrate NTs. 2, The relationships of the ancient NTs to the vertebrate NTs and the insect clades varies with the trees, particularly in the case of acorn worm (SkNT). Structural alignment had also revealed a closer similarity of SkNT to DNT1 and Spz as well as the vertebrate NTs than BfNT or SpNT. 3, Although Maximum Likelihood is the best method for distantly related sequences, the low bootstrap values in (E) indicate that sequence divergence is too high to resolve the phylogeny.

Figure S4 DeadEasy software for the automatic quantification of apoptosis in vivo. (A) Anti-cleaved-Caspase-3 (Caspase) is a reliable apoptotic marker. Co-detection of the apoptotic markers TUNEL (magenta) and Caspase (green) in a single 0.5μ section of a stained embryonic VNC. Single channel higher magnification details of one cell are shown on the right. (B) How DeadEasy software quantifies cells. We wrote DeadEasy as an Image-J plug-in. Whole embryos are stained in vivo with α-Caspase-3 and the whole thickness of the ventral nerve cord (VNC) is scanned under the confocal microscope, sections are 0.25m apart, over 100 sections per VNC. A Region Of Interest (ROI) is drawn over the lateral edges of the VNC to eliminate epidermal apoptosis from the counts. DeadEasy is run as an Image-J plug-in throughout the whole stack. Each individual section is processed to identify objects. Identified cells are labelled throughout the stack and they are classified first in 3D according to minimum volume and also based on minimum pixel intensity. DeadEasy produces a message with the total number of α-Caspase-3 cells counted in about one minute per embryonic VNC (or stack). For details see Supplemental Procedures.

Figure S5 Spz and DNT2 orthologues in insect species. Alignment of the Cysknot domain of (A) Spz and (B) DNT2 to their orthologues from insects with sequenced genomes, including 12 Drosophila species, three mosquito species (Anopheles aegypti, A. gambiae and Culex pipiens), beetle (Tribolium castaneum), sylkmoth (Bombyx mori) and human body louse (Pediculus humanus corporis). Identical residues are shown in white over red; conservative substitutions in red. There is conservation of Spz and DNT2 in insects within the Cysknot, lower for Spz. For accession numbers see Supporting Methods 1.4.

**Figure S6 Muscles develop normally in DNT1 and DNT2 mutants.** (A) Anti-Myosin stage 17 stained embryos, three different focal planes are shown from top to bottom. Arrows point at muscles shown in each focal plane and which coincide with the expression domains of DNT1, DNT2 and Spz. No muscles defects were observed in stage 17 stained embryos. Some stage 13-16 *spz^2^* and *DNT2^e03444^* mutant embryos have abnormal morphology and CNS defects, and the penetrance of these abnormal embryos can increase to 20-40% in the double and triple mutant embryos. These severe phenotypes might be a consequence of earlier developmental defects in dorso-ventral patterning, as they can be seen prior to muscle development. To ensure that only zygotic functions are analysed, we focus on stage 17 embryos. (B) Targeting defects occur independently of muscle defects: here three different focal planes are shown to indicate normal muscle patterning with loss of axonal targeting. Arrowheads indicate muscles, arrows axons. There are occasional muscle defects at stage 17 particularly in triple mutant embryos. Thus it is possible that DNTs may also play functions in the muscle. Axon guidance and targeting phenotypes can be dissociated from muscle phenotypes.

**TEXT S1**

**SUPPORTING MATERIALS AND METHODS**

***Bioinformatics***

***Identification***

Full length and Cystine knot sequences from 28 known vertebrate NTs were used in Gapped-BLAST (TBLASTN) and PSI-BLAST searches (<http://www.ncbi.nlm.nih.gov/blast/blast.cgi>) against release 2 of the Drosophila genome. Sequences were aligned using ClustalW (www.ebi.ac.uk/clustalw/), Pileup ([www.gcg.com](http://www.gcg.com/)) and Pfam ([www.sanger.ac.uk/Software/Pfam/](http://www.sanger.ac.uk/Software/Pfam/)). Carp *(C.carpio)* BDNF showed homology with CG18318 both in BLAST and PSI-BLAST searches as the only hit in Drosophila, with all 6 Cysteines conserved. This hit was verified by reverse-BLAST. When CG18318 was queried against the Swiss-Prot non-redundant database (http://expasy.org/sprot/), it identified BDNF sequences from at least 8 different organisms, including human BDNF. Isolation and sequencing of cDNA3 (which encodes DNT1, GenBank accession number: FJ172423) demonstrates that CG18318 identified in release 2 corresponds to CG32244 plus CG32242 from release 3, which therefore belong to the same locus. When the DNT1 protein sequence of cDNA3 was used as query in PSI-BLAST searches to the Swiss-Prot non-redundant database, it identified as top hits BDNF from multiple fish species, including bastard, halibut, carp, zebrafish and platyfish. BLAST to the human ENSEMBL database (<http://www.ensembl.org/>) using DNT1 as the query sequence identified BDNF.

FUGUE was developed[2] to identify distantly related proteins, the amino-acid sequence of which may have diverged despite structural conservation (http://www‑cryst.bioc.cam.ac.uk/fugue/). The program searches the HOMSTRAD[3] (www-cryst.bioc.cam.ac.uk/homstrad/) database of proteins of known structure by using substitution matrices, where the scores for amino acid substitutions are determined depending on how they affect protein secondary or tertiary structure. When DNT1 is used as a query, FUGUE identifies with over 99% certainty the human neurotrophins, comprising BDNF, NGF, NT3 and NT4 as homologues. Coagulogen is identified with 95% certainty, thus DNT1 is less similar to Coagulogen than to NTs.

DNT1 shares with other ancient NT a longer pro domain (as in Lamprey Lf-NT and sea urchin Sp-NT) and an extended COOH tail (as in Amphioxus Bf-NT and sea urchin Sp-NT) compared to canonical vertebrate NTs.

***Structural alignment and model of the DNT1 protomer***

To verify the homology of DNT1 to NTs we carried out structural alignments, making use of the advantages offered by FUGUE. The amino-acid sequences of neurotrophins and insect orthologues of DNT1 were retrieved from the National Center of Biotechnology Information ([www.ncbi.nlm.nih.gov](http://www.ncbi.nlm.nih.gov/)): lamprey NT (Lf-NT; AF071432[4]), Amphioxus NT (Bf-NT; DQ447321[5]), sea urchin NT (Sp-NT1; DQ447322[5]), Anopheles gambiae PEST DNT1 (AgNT1; ENSANGP00000029467), and Drosophila pseudoobscura NT1 (DpNT1; GA16782-PA). The amino acid sequence of the acorn worm Cystine-knot (Sk-NT) was retrieved from Hallböök et al.[5]. The Cysknot domain corresponds to residues: 170-324 for Lf-NT, 128-244 for Bf-NT, 266-374 for Sp-NT, 15-113 for AgNT1 and 47-145 for DpNT1. The full sequence of Sk-NT is not published. These sequences for the Cysknot domains were aligned against the HOMSTRAD[3] entry of the nerve growth factor (NGF) family using FUGUE[2]. The NGF family consists of four proteins of known three-dimensional structure: human BDNF (hBDNF; Protein Data Bank [PDB: <http://www.wwpdb.org/>] 1bnd[6], chain A), human NT3 (hNT3; PDB 1bnd[6], chain B), human NGF (hNGF; PDB 1bet[7]) and human NT4 (hNT4; PDB 1b98 chain M[8]). FUGUE uses the structural information to optimize the sequence-structure alignment. The resulting alignment was visually examined and adjusted by hand. The final alignments (Figure1B and Figure S5) were formatted with ESPript 2.2[9] (http://espript.ibcp.fr/ESPript/ESPript/).

This multiple sequence alignment suggested hBDNF as the best template for modelling the structure of DNT1. The sequences of only DNT1 and hBDNF were extracted and adjusted further by hand. Using this alignment, a model of DNT1 was built

with MODELLER[10]. The schematic drawing of the model (Fig.1D) was produced with PyMOL (www.pymol.org; blue; N-terminus, red; C-terminus). The predicted disulphide bridges were shown as stick-and-ball model.

# *Phylogenetic analysis*

Phylogenetic analysis was attempted using sequences comprised with the Cysknot domain only, as sequences diverge considerably outside the Cysknot. Sequence identity within the Cysknot between the Drosophila and vertebrate sequences is <30%. The structural alignment and sequence ranges described above were used to perform the phylogeny. Phylogenetic trees were generated using Phylip v3.67 (downloadable from

<http://evolution.genetics.washington.edu/phylip.html>) following three different methods (Phylip programs in brackets): (1) Neighbour-joining (Seqboot - Protdist - neighbour - consense - drawgram); (2) Maximum parsimony (Seqboot - Protpars - consense - drawgram), and (3) Maximum likelihood (Seqboot - Proml - consense - drawgram). In all cases, 1000 bootstraps were performed using the Jones-Taylor-Thornton matrix[11]. In order to randomise the input order of sequences every time a tree was generated, the random number seed 2113 was used, and sequences were jumbled 7 times per bootstrap, to allow sufficient mixing of sequences over the 1000 bootstraps. A consensus of the 1000 trees per method was generated using majority rule, and the trees were rooted using Coagulogen from Horseshoe crab (Limulus polyphemus, accession: X04424).

***Identification of DNT1, Spz and DNT2 insect orthologues***

The BLAST server at FlyBase (http://www.flybase.org/blast/) was used to identify

orthologues of DmNT1, DmNT2 and DmSpz in other insect species (see also: http://rana.lbl.gov/drosophila/). We searched all sequenced genomes. Sequences comprising the Cysknot were used in TBLASTN searches of GenBank and Genomic Scaffold sequences. To verify homologous candidate genes, reverse-BLAST was performed by using the candidate sequence to search the *D.melanogaster* genome. In all cases, DmNT1, DmNT2 or DmSpz, respectively, were found as the top hit. The alignments were generated in Clustalw v1.83, using the *D.melanogaster* sequence as a scaffold to which the homologues were aligned.

We found DNT1 orthologues in all sequenced insect species except red flour beetle (Tribolium castaneum). We found a Spz orthologue in T.castaneum but we did not find Spz orthologues in malaria mosquito (A.gambiae), jewel wasp (N.vitripennis) and human body louse (P.humanus corporis). Thus DNT1 is more conserved than Spz. The following orthologues followed by species name and accession numbers were identified: From fruit-flies, Drosophila species with GLEANR accession numbers from <http://rana.lbl.gov/drosophila/> : (1) *D. ananassae* DNT1 9428; Spz 7494; DNT2:10234; (2) *D. yakuba* DNT1 4440; Spz 10503; DNT2: 21683; (3) *D. sechillia* DNT1 14779; Spz 14443; DNT2: 14097;(4) *D. simulans* DNT1 13298; Spz 1927; DNT2: 13369; (5) *D. willistoni* DNT1 17888; Spz 12196; DNT2: 17627;(6) *D. erecta* DNT1 14373; Spz 12191; DNT2: 14494; (7) *D. persimilis* DNT1 19785; Spz 5951; DNT2: 22428;(8) *D. psuedoobscura* DNT1 7992; Spz 4177; (9) *D. grimshawi* DNT1 15556; Spz 3285; DNT2: 14578; (10) *D. virilis* DNT1 15915; Spz 9440; DNT2: 11759; (11) *D. mojavensis* DNT1 12583; Spz 8429; DNT2: 13903. From mosquito: (1) African malaria mosquito, *Anopheles gambiae* accession numbers for orthologue of DNT1: AAAB01008960; DNT2: CM000356;(2) yellow fever mosquito, *Anopheles aegypti*: orthologues of DNT1: CH477217.1; Spz: CH477194.1; DNT2: CH477231; (3) southern house mosquito, *Culex pipiens quinquefasciatus*, DNT1: DS231814.1; Spz: DS232347.1; DNT2: DS23062; (4) honey bee, *Apis mellifera*, orthologue of DNT1: AADG05004611.1; (5) jewel wasp, *Nasonia vitripennis*, orthologue of DNT1: AC185338.4; (6) human body louse, *Pediculus humanus corporis* orthologue of DNT1: DS235024.1; DNT2: DS235882; (7) red flour bettle, *Tribolium castaneum*, orthologue of Spz: CM000277; DNT2: CM000280; (8) sylkmoth, *Bombyx mori*: orhtologue of DNT2: BAAB01047359.

***DNT1 cleavage prediction***

Cleavage prediction analysis using the ProP server (<http://www.cbs.dtu.dk/services/ProP>) reveals two high scores at positions 283 and 294. However, the sequence most likely to match the cleavage site of Spz by Easter is FSLSKKR RE at position 498. Although the scoring for this site is low (p=0.278), this could be a false negative, as the ProP scoring for the known Easter cleavage site for Spz is also low at p=0.202. This suggests that position 498 is a candidate for cleavage of DNT1. There are also candidate cleavage sequences downstream of the Cysknot: CQVDGYR QQ at position 601 and LSSIQAK DY at position 613.

*Search for DNT1 homologues in the cnidarian Nematostella and mollusc Aplysia*

We searched for sequence homologues of DNT1 in the sequenced genome of Nematostella vectensis and the EST collections of N.vectensis and Aplysia californica, at the following databases: (1) [www.ncbi.nlm.nih.gov/sites/entrez/](http://www.ncbi.nlm.nih.gov/sites/entrez/); (2) //aplysia.cu-genome.org/cgi-bin/blast; (3) //evodevo.bu.edu/stellabase/. We used the DNT1 protein sequence, either the Cysknot or the full-length, to query the Nematostella genome and both EST databases using PSI-BLAST and TBLASTN. We also used the Cysknot from Spz, other ancient NTs (Bf-NT, Sp-NT, Sk-NT), carp and human BDNF and human NGF. Candidate hits were checked by reverse-BLAST and by FUGUE. No candidate sequences were confirmed by these two approaches thus we conclude that no DNT1 or Spz homologues were found in Nematostella or Aplysia.

***Genetics***

***Mutant alleles:*** i) Null: *DNT1^41^ and DNT1^55^* (generated in this work); ii) *Df(3L)ED4342/TM6BlacZ*. Mutant analysis was done using homozygous *DNT1^41^* mutant embryos and transheterozygotes of genotype *DNT1^41^/DNT1^55^*and *DNT1^41^/Df(3L)ED4342*. ii) The allele *spz^2^/TM6BlacZ* (Bloomington) - a point mutation in the pro-domain – was chosen because it is semi-lethal (7.5% escapers), which indicates that maternal product is sufficient to enable normal early development and it favours the analysis of genetic interactions. Mutations in the pro-domain of Spz affect normal function[12] and in BDNF they reduce overall BDNF[13] levels by preventing secretion. iii) Toll alleles: *Toll^r3^/TM6BlacZ* and *Df(3R)ro80b/TM6BlacZ*, both are embryonic lethal Both *spz* and *Toll* mutants have maternal contribution. We use here only zygotic null embryos (from heterozygous mothers) and we assume that maternal contribution has decreased or been lost by the end of embryogenesis. A fraction of homozygous *spz^2^* stage 13-16 embryos have a severe phenotype and were not analysed here, as they may correspond to secondary consequences of loss of Spz during early embryogenesis. Stage 17 embryos are morphologically normal. . iv) DNT2 mutants: DNT2^eo3444^ is a PiggyBac insertion into an intron, and Df(3L)Exel6092/TM6BlacZ uncovers the DNT2 locus. v) Double mutants: *spz^2^* *DNT1^41^/TM6BlacZ* , *DNT2^eo3444^ DNT1^41^/TM6BlacZ* and *Df(3L)Exel6092 DNT1^41^/TM6BlacZ* and triple mutants *DNT2^eo3444^ DNT1^41^ spz^2^* were generated by conventional genetics. In the double and triple mutants, between 20*-*40% embryos have abnormal morphology at stages 13-16 and were not analysed here, as they could correspond to incomplete rescue of maternal loss of function by the zygotic products. Homozygous mutant embryos were identified by staining balancer embryos with anti-βgal antibodies.

***Gain of function and targeted RNAi***

The following GAL4 lines of flies were crossed to the UAS lines below: **GAL4 drivers**: i) *simGAL4/CyO; simGAL4/simGAL4* drives expression at the embryonic midline; ii) *24BGAL4* drives expression at the muscle; iii) *elavGAL4* drives expression in all neurons; iv) *6099GAL4* drives expression at the lamina. **(4) UAS lines:** generated as described below and in 3.2, were mapped and homozygosed by conventional genetics and crossed to the above GAL4 drivers. **For targeted RNAi** we used: For **RNAi to the Cystine-knot** domain, which down-regulates only cDNA3 with the Cysknot: i) Two copies of Cysknot RNAi: w;UASCysknotRNAiC; UASCysknotRNAiB. RNAi in trans to DNT1 mutations: ii) w;UASCysknotRNAiC; Df(3L)ED4342/TM6BlacZ; iii) w;UASpwCysknotRNAi; Df(3L)ED4342/TM6BlacZ; iv) w;UASpwCysknot; DNT1^41^; v) w;UASCysknot; DNT1^41^. For **RNAi to the pro-domain**, which downregulates all DNT1 transcripts, including cDNA3 bearing the Cysknot: v) w;UASpwcDNA1RNAi; Df(3L)ED4342/TM6BlacZ; vi) w;UASpwcDNA1RNAi; DNT1^41^. **For gain of function** we used: i) Full length: w;UAScDNA3-EGFP (GenBank: FJ172423); ii) Pro domain: w;UAScDNA1 (GenBank: BK006739), which comprises most of the pro-domain up to residue 454aa of cDNA3; iii) Cysknot: w;UASCysknot; iv) w;UASCysknot3’tail; v) w; UASDNT2 Cysknot. UASactivatedSpz corresponds to the Spz Cysknot only, and UASactToll^10b^ to activated Toll (both gifts from J.M.Reichhart and T.Ip). In all stainings (where applicable) mutant specimens were identified by the lack of TM6BlacZ balancers, stained with anti-βgal antibodies.

***Homologous recombination***

Null alleles for DNT1 were generated by homologous recombination using the ends-out protocol. The coding region of DNT1 including the ATG and the whole Cysknot domain, was replaced by the coding region of the *white* gene. This was done first by generating transgenic flies that had the *white* gene (thus red eyes) flanked by sequences homologous to 5’ and 3’ ends of DNT1 (called 5’ and 3’ arms). This insert was then liberated from the genome resulting in white-eyed flies, and induced to recombine as a free fragment with the endogenous DNT1 locus. When homologous recombination takes place, flies in the next generation have red eyes. 4841 bp (gene sequences 195458-200271 of GenBank accession number AE003481) upstream of the DNT1 ATG were PCR-amplified from genomic DNA to use as the 5’ arm (5’ primer: AGGCGCGCCTGAATATTACACCATTTGAATGCT and 3’primer: GCCCTAGGGCTGCAGTCGTCTGCGAATGACA). 4539bp (gene sequences 188341-192880 of Gene Bank access number AE003481) downstream of the Cysknot domain and spanning the neighbouring gene CG13720 were PCR-amplified to use as the 3’ arm (with 5’ primer: GACGGTCCGGTGGATGGTTATCGCCAGCA and 3’ primer: TCCCCGCGGGAGGAGTTGAGGCGAAGAGT), and both fragments were inserted into the CMC105 plasmid (gift from G.Struhl and L. Vosshall), flanking the *mini-white* gene and between P-element inverted repeats. The resulting 18kb construct was injected into *yellow white* Drosophila embryos. Seven lines of transformant flies were homozygosed and red-eyed homozygous virgin females were crossed in groups of 5 to w;P70FLP SceI Sco/CyO males, embryos were heat-shocked after three days in a water bath at 38° for 1.5hr. F1 white eyed virgin CyO+ females were selected and crossed in groups of 5 to w;P70FLP males, embryos where heat-shocked after three days as above. F2 red-eyed virgin females or males were selected, crossed to the TM6B balancer and stocks established. Out of 8,500 F1 females crossed we established 64 red-eyed insertion lines, out of which 2 were homologous recombinants. Lines DNT1^41^and DNT1^55^ lack the translation start codon and pro- and Cysknot domains. Sequencing of the entry points in which the recombined sequences link to the genome revealed that DNT1^41^ and DNT1^55^ are molecularly identical, as expected, although they are most likely the result of independent events and therefore not necessarily genetically identical.

***Cloning and transgenesis for gain of function and RNAi experiments***

***Isolation of full length cDNA3***

The DNT1 locus corresponds to CG18318 from release 2 and CG32244 plus CG32242 from release 3 of the sequenced genome (www.flybase.org). We identified 4 transcripts encoded in the DNT1 locus (Fig.1C). cDNA1 (GenBank: BK006739) corresponds to RC and cDNA2 (GenBank: BK006740) to RB from CG32244 [www.flybase.org](http://www.flybase.org/).; cDNA4 (GenBank: FJ172424) differs from cDNAs1 and 2 only in the 5’ untranslated region; cDNA3 (GenBank: FJ172423) spans cDNA1/2 (CG32244) and the Cysknot domain encoded by CG32242. The existence of cDNA3 transcripts was verified by RT-PCR on RNA from embryos, larvae and adult heads using 5’ primers from the 3’ of cDNA1 (CG32244, primer: 5'-AGTTTGCGGCGAGTGTTGAAA-3') and 3’ primers downstream of the Cysknot domain (CG32242, primer: 5'-TTGCTAGAACTCTCGTGACA-3') (Fig.S2B). Full length cDNA3 was amplified by PCR from cDNA libraries (embryonic: LD, larval/pupal: LP, adult head: GH from DBGP [www.fruitfly.org](http://www.fruitfly.org/)) using primers to the 5’ and 3’ predicted untranslated regions of CG18318 (at the 5’ UTR: 5'-AGTTTGCGGCGAGTGTTGAAA-3' and at the 3’ UTR: 5'-TTGCTAGAACTCTCGTGACA-3' (Fig.S2B). DNT1 (cDNA3) was sequenced and presents the following characteristics: MW 100kDa, PI:6.17. DNT1 is 886 aa long, with a Signal Peptide (1-29aa), a pro-domain (30-498aa), a 102aa Cysknot domain (499-600aa) and an extended, disordered 286aa COOH tail (601-886aa).

***Transgenesis***

***UAS constructs for ectopic expression:*** we inserted coding sequences from DNT1 into the EcoRI and XbaI sites downstream of UAS in the pUASt vector: DNT1 Signal Peptide (residues 1-30aa amplified with primers: forward 5'-CGGAATTCAAAATGAAAGCTGGCCGCG-3' and reverse 5'-CCGCTCGAGCTCATCGTCGGCGGAGTT-3’) was flanked by EcoR1 and XhoI sites and was followed by: the Cysknot (residues 499-601aa of Full-length cDNA3); Cysknot3’tail ( 499-886aa of full-length cDNA3); cDNA1 ( residues 1-454aa); cDNA3 (residues 1-886aa ) flanked by Xho1 and XbaI sites. These were injected into *yw* embryos together with a plasmide encoding *transposase*, for conventional Transposase-mediated germline transformation. For DNT2 (CG9972 previously named *spz5,* GenBank: AAK93159), the DNT2 Signal Peptide (residues 1-32aa amplified from EST LD26258 with primers forward 5’-CGGAATTCATGCAAATCGACGGCGAATGA-3’ and reverse 5’-GAAGATCTCGAGCTGTGGGCGGCTACTGT -3’) was inserted into pUASTattB sites EcoRI and BglII followed by the Cysknot domain (residues 281-387aa amplified with forward primer 5’-GAAGATCTAGGACAAAGCGCCAAAGTCC -3’and reverse primer 5’-GCTCTAGATTCGTCCTTCGTCCGCTGGA-3’) flanked by BglII and XbaI sites. The resulting construct was injected into J35 PHI-X-86Fa flies for PHI-31 convertase mediated transformation[14].

***Targeted UAS-RNAi constructs:*** The following sequences were used for RNAi constructs: (1) Cysknot-RNAi: two copies of a fragment encoding the Cysknot domain of DNT1 (1910-2357bp of full-length cDNA3) were inserted in opposite orientations separated by an endogenous intron of DNT1, into the pUASt vector (between EcoRI and XbaI sites). (2) pWIZCysknot-RNAi: 574bp fragment of cDNA3 (2057-2630bp) including the Cysknot domain. This sequence overlaps in 300bp with the above Cysknot RNAi (2057-1357bp) and it differs from it in 274bp (1910-2056 and 2358-2630bp). (3) Pro-domain RNAi: pWIZcDNA1RNAi fragment (370-872bp of cDNA3) included in the pro-domain and which does not overlap with the Cysknot fragments. The pWIZ vector (provided by DGRC) was designed for enhanced targeted RNAi, and it is pUASt derivative with a 74bp second intron from the *white* gene downstream of UAS and flanked by two multi-cloning sites, into which the sequences of choice were inserted in opposite orientations (at EcoRI, AvrII, NheI and XbaI sites). The resulting transformant flies were mapped, established as stable homozygous stocks and crossed to appropriate GAL4 drivers (see above).

*RT-PCR and Southern blots for verification of RNAi and Homologous recombinants*

RT-PCR was used to verify that targeted RNAi in a heterozgygous mutant background resulted in a down-regulation of DNT1 transcripts encoding the Cysknot. Under the same conditions, the null DNT1^41^ mutants do not produce transcripts, whereas heterozygote embryos produce transcripts in normal levels. The following primers were used for RT-PCR data in Figure 5C: Forward: 5’-TAGACGACTGCAATTAAAAACAA-3’ and reverse: 5’-GCCTGTTCAAACTTTAGTTCGT-3’. Southern blot was used as a further proof of homologous recombination to show a band shift at the location where the *white* gene replaced the coding region of DNT1. The blot was hybridised with a probe spanning 1774bp of cDNA3, i.e. Cysknot plus 3’tail (positions 1978-3752).

***Cell culture transfections and western blotting***

Cell culture and western blotting were used to verify cleavage and dimerisation of DNT1. S2 cells were grown and transfected in Schneider Drosophila medium (Invitrogen) following standard procedures with the following inserts into the EcoRI and NotI sites of the Drosophila expression vector pAc5.1/V5-His, and upstream of the V5 tag: 1) cDNA3 up to position 716aa; 2) Signal-peptide-CysKnot (SP: 414-516bp; Cysknot: 1866-2219bp of cDNA3); 3) Signal-peptide-Cysknot3’tail (SP: 414-516bp; Cysknot3’tail: 1866-3077bp of cDNA3). The transfected cells were harvested and lysed for Western blotting and V5-tagged DNT1, Cysknot3’tail or Cysknot were detected with anti-V5 antibody (1:5000, Invitrogen). 2.5% β-Mercaptoethanol was added to all samples for reducing conditions.

***In situ hybridisations and immunohistochemistry***

These methods were carried out following standard protocols, except that for Toll stainings embryos were fixed for 10 minutes. In situ hybridisations were performed with RNA probes to visualise the expression of DNT1 and *spz*. For DNT1, a DNT1-Cysknot specific RNA probe was made from a pBS-Cysknot plasmid, after linearization with XbaI and transcription with T7 RNA polymerase. A probe that detects all isoforms was made from cDNA1 in a pFLC-1 plasmid, after linearization with Not1 and transcription with T7 RNApol. *Spz* probes were made from the LD02813 EST from DGRC, by linearising with BamHI and transcribing with T7 RNA polymerase. DNT2 probes were made from EST LD26258 in plasmid pOT2, after linearising with BglII and by transcribing with SP6 RNA polymerase. TUNEL was carried out to visualise apoptotic cells using d-12-UTP-FITC following standard procedures[15]. Antibody dilutions were as follows: (1) Rabbit anti-cleaved-Caspase-3 1:50 (Upstate); (2) Mouse FasII (ID4) 1:5; (3) Mouse Eve 1:5 (Iowa HB); (4) Guinea-pig HB9 1:1000 (gift of H.Broihier); (5) Mouse 22c10 1:5 (Iowa HB); (6) Mouse anti-βgal 1:1000 (Sigma); (7) Rabbit anti-βgal 1:2500 (Cappel); (7) Rabbit anti-Toll 1:200; (8) Rabbit anti-Myosin 1:500. Secondary antibodies were directly conjugated to Alexa-488, Alexa-546 and Alexa-660, or biotinylated followed by Streptavidin-Alexa. Samples were mounted in Vectashield or 70% glycerol. Anti-Caspase3 was detected with Rabbit Alexa- 488 without further amplification.

***Microscopy***

Confocal microscopy was carried out with BioRad Radiance 2000 and Leica SP2-AOBS laser scanning confocal microscopes. For automatic quantification using DeadEasy, Caspase-3 was scanned using the Radiance 2000 confocal microscope, see details below. Colocalisation of Caspase-3 and fluorescent HB9, Eve, FasII and 22c10 was scanned with the Leica SP2 at sections 0.5μm apart, 2x averaging, 63x, zoom 1.6. For Myosin, images were acquired at 0.5μ sections and with a 40x lens. Wide-field microscopy was done in a Zeiss Axioplan 2 microscope, JVC camera and Neotec Image Grabber card.

***Informatics resource: DeadEasy software for the automatic quantification of apoptotic cells***

We purposely wrote the DeadEasy software in Java as an ImageJ plug-in, to quantify automatically cells stained with the apoptotic marker anti-cleaved-Caspase3 (Forero, Pennack, Learte and Hidalgo, in preparation). Antibody staining conditions were optimised to minimise background by placing very small volumes of embryos (10μl) in an incubation volume of 50 μl. The antibody was not re-used. The data are collected as stacks of confocal sections with voxel size 250μm^3^, magnification 60x, zoom 1.6, with no averaging and resolution 512x512 pixels. A region of interest (ROI) is selected over the embryonic CNS Ventral Nerve Cord to exclude the epidermis and all ventral tissues. The software identifies stained cells in each individual section and subsequently in 3D, based on minimum area, volume and pixel intensity. Following object recognition, DeadEasy creates a new stack of images equivalent to the orignal confocal stack, where each identified cell is visualised mimicking the raw data in shape, size and position. Each identified cell has a unique identifier: by placing the pointer over the cell a number is revealed and one can check if adjacent cells are counted as one or two cells. The software was verified using 18 stacks of confocal sections from independent embryos and experiments, making up a total of 1,954 Caspase positive cells. Each of the cells was verified manually in individual sections by comparing the raw data with the DeadEasy processed stacks. Verification of the software estimates for DeadEasy 1.01% false positives (counted cells that are not apoptotic) and 1.98% false negatives (missed apoptotic cells) (n=1,954) and sensitivity = 0.98%. Sensitivity is a measure of how well our binary classification test correctly identifies an object (Caspase cell), = 1 is the maximum. As a negative control, H99[16] mutant embryos (which lack apoptotic cells) were used: no positive cells were detected by DeadEasy in 3 stacks of confocal sections of 3 different H99 mutant embryos). DeadEasy counts the total number of apoptotic cells per stack in about one minute. Mathematical algorithms used to write the software will be published elsewhere (Forero et al, in preparation).

# *Statistical analysis*

***Automatic quantification of Caspase3-positive cells with DEADEASY***

Caspase-3 positive cells in Figures 4B-E, and Fig8D are quantified automatically with DeadEasy software (see Supporting Methods8 above and Figure S4). The mean number of apoptotic cells stained with anti-cleaved-Caspase-3 per embryo and per genotype is given in bar graphs, sample size n are given above the bars and n= number of embryos (therefore VNCs) analysed. To test the significance of the differences between the means, we first tested the normality of the distributions by measuring the skewness and/or kurtosis, and the homogeneity of variances with a Levene test. Accordingly, all the distributions in Fig.4e and Fig.7 are normal and variances equal, except for samples: DNT1^41^/DNT1^41^, ElavGAL4/UASDNT1FL and simGAL4 UASDNT1 CK where skewness and/or kurtosis depart from normality However, since they only marginally do so and sample sizes are not large, we assume that these distributions tend to normality as for all other genotypes. The null hypothesis is that the extent of apoptosis in experimental genotypes does not differ from that of controls. We tested this with a One-way ANOVA for each set of experiments and a Student-t test was then used to compare the means between selected genotypes (i.e. to wild-type or an appropriate control). CI is 95%, P values for Figure B-E are given as follows. **DNT1 rescues NOCD** (GOF, gain of function) (One-Way ANOVA: F=19.230 p=0.000): There is a significant reduction in the mean number of apoptotic cells at stage 13/14 upon expression in all neurons of Cysknot (Student t-test: CK vs wt: t: p=0.000) and upon expression of Cysknot3’tail (CK3+ vs wt: t: p=0.000) compared to wild-type. **DNT1 is in limiting amounts** (One-Way ANOVA: F=30.425, p=0.000): There is a significant reduction in the mean number of apoptotic cells at stage 13/14 upon the expression at the midline of Cysknot (CK vs wt: t: p=0.01) and of Cysknot3’tail (CK3+ vs wt: t: p=0.000) compared to wild-type. **DNT1 mutants**: there is a significant increase in the number of apoptotic cells at stage 17 in loss of function for DNT1 mutant embryos (One-Way ANOVA: F=12.831 p=0.000): in DNT1^41^ homozygous mutants (DNT1^41^ vs wt, t: p=0.000), in trans-heterozygotes over DNT1^55^ (DNT1^41^/ DNT1^55^ vs DNT1^41^/+ t: p=0.000) and over the deficiency Df(3L)ED4342 uncovering the DNT1 locus (DNT1^41^/Df(3L)ED4342 vs DNT1^41^/+ t: p=0.01). **Rescue**: there is a significant decrease in the mean number of apoptotic cells at stage 17 upon expression of the DNT1 Cysknot in all neurons in DNT1^41^ mutant embryos (DNT1^41^ ElavGAL4/ DNT1^41^ UASCK vs DNT1^41^ /DNT1^41^, t: p=0.000). **DNT1 is required in target cells**: there is a significant increase in the number of apoptotic cells throughout the nerve cord upon targeted RNAi to the midline (One-Way Anova: F=4.88 p=0.000). There is a significant increase in apoptosis throughout the nerve cord upon targeted pro-RNAi to the midline in a DNT1^41^ heterozygote background compared to a control expressing pro-RNAi in a wild-type background (simGAL4/UASpwcDNA1RNAi DNT1^41^ vs simGAL4/UAScDNA1RNAi t, p=0.000) and compared to a DNT1^41^ heterozygote control (simGAL4/UASpwcDNA1RNAi DNT1^41^ vs DNT1^41^/+ t, p=0.006). There is a significant increase in apoptosis throughout the nerve cord upon expressing at the midline Cysknot-RNAi compared to a heterozygote control (simGAL4/UASCysknotRNAi; DNT1^41^ vs DNT1^41^/+ t: p=0.01). Figure 8D: **Activated Toll and activated DNT2 rescue NOCD** (One Way Anova: F= 3.557 p=0.03): There is a significant reduction in the mean number of apoptotic cells at stage 17 upon expression in all neurons of activated Toll (*elavGAL4/UAS* actToll vs wt, t: p=0.02) or activated DNT2 (*elavGAL4/UAS DNT2 Cysknot vs wt: p=0.02)* **Apoptosis in *spz^2^* mutants**: (One Way ANOVA: F= 7.074 p=0.000). There is a significant increase in the mean number of apoptotic cells in loss of function *spz^2^* mutant embryos (*spz^2^* vs wt t: p=0.002), Toll mutant embryos (Toll^r3^/Df(3R)ro80b vs wt t: p=0.027) *spz^2^* *DNT1^41^* (double mutants and compared to wild-type (*spz^2^* *DNT1^41^* vs wt t: p=0.001). There is no significant difference in the extent of apoptosis in *spz^2^* *DNT1^41^* double mutant embryos compared to single mutants (*spz^2^* *DNT1^41^* vs *DNT1^41^* t: p=0.280). There is a significant reduction in apoptosis upon expression of activated Spz in *DNT1^41^* mutants (*DNT1^41^ ElavGAL4/UASactSpz DNT1^41^ vs DNT1^41^/DNT1^41^* t: p=0.03). **Apoptosis in *DNT2* mutants and *DNT1 DNT2* double mutants:** there is a significant increase in apoptosis in *DNT2* mutant embryos and *DNT1 DNT2* double mutant embryos compared to wild-type (One Way ANOVA: p=0.000). There is a significant increase in apoptosis in *DNT2* mutant embryos compared to wild-type (*DNT2^e03444^/Df(3L)6092* vs. wt t: p=0.03). There is significant increase in apoptosis when comparing DNT1 Dnt2 double mutants with DNT2 single mutants (*DNT2^e03444^/Df(3L)6092* vs *DNT2^e03444^ DNT1^41^/Df(3L)6092 DNT1^41^* t: p=0.001).

***Manual quantification of interneuron and motorneuron apoptosis***

***Colocalisation of HB9 with anti-cleaved-Caspase-3****.* Fig.5a. Colocalisation was quantified manually by checking every confocal stack one section (0.5μm apart) at a time, in separate channels as well as merged, and comparing it with the whole stack. We counted the number of colocalising cells expressing both HB9 and Caspase3 per segment, within a population of embryos, for each genotype (we used segments rather than hemisegments because some motorneurons are located at the midline). dMP2s are excluded because they undergo cell-autonomous apoptosis in wild-type. Half of the segments in wild-type embryos have 0 or 1 apoptotic cell only, thus the graph shows the division of samples into two categories: percentage of segments with ≤1 and with >1 apoptotic cell. Relative frequency is used to compare genotypes. We asked whether these categories differ significantly with a  for planned comparisons. CI 95%, P values are: There is a significant increase in the number of segments with more than one apoptotic neuron in the CNS of: *DNT1^41^ /DNT1^55^* mutant embryos (χ^2^ p=0.05) and in embryos transheterozygote for *Df(3L)ED4342* and *DNT1^41^*  (*DNT1^41^/Df(3L)ED4342* χ^2^ p=0.002). The distributions of the raw data are not normal (not shown). The distributions differ significantly, as verified using the non-parametric Kruskal-Wallis H test for multiple comparisons (H: χ^2^ 13.248, p=0.01). To verify if the distributions of experimental loss of function samples differed from the wild-type control we carried out planned comparisons using the Mann-Whitney U test. There is a significant increase in the number of cells colocalising HB9 and Caspase3 in: *DNT1^41^ /DNT1^55^* mutant embryos (U p=0.05) and in embryos transheterozygote for *Df(3L)ED4342* and *DNT1^41^ (DNT1^41^/Df(3L)ED4342* U p=0.004).

***Colocalisation of Eve with anti-cleaved-Caspase-3***

Fig.5B: Quantification was carried out manually by checking every confocal stack one section (0.5μm apart) at a time, in separate channels as well as merged, and comparing it with the whole stack. Histograms show manual quantification of the colocalisation of Eve and Caspase-3 in ELs and U/CQs identified based on their location (aCC, pCC and RP2 are excluded from quantification because they undergo apoptosis in wild-type), sample sizes n given on figure. There is a significant increase in the number of embryos with more than 1 apoptotic U/CQ motorneuron in DNT1^41^ mutants (DNT1^41^ vs wt, χ^2^ p=0.02) and more than 1 apoptotic EL interneuron (DNT1^41^ vs wt, χ^2^ p=0.05, planned comparisons).

***Loss of Eve positive cells:*** Fig.6C: There is a significant loss of Eve positive EL and U/CQ neurons per segment in loss of function for DNT1 (DNT1^41^ /DNT1^55^ vs wt, t: p=0.000), n= number of segments given on figure. In all experiments expression in all neurons is with *elavGAL4*, at midline with *simGAL4* and in the muscle with 24BGAL4. Only abdominal segments A1-A7 are scored.

***Axonal phenotypes*** Figure 7 and Figure 9. Phenotypes of the ISNb/d and SN projections are subdivided in qualitative classes. Only misroutings in ISNb/d and in SNa and effects in more than one projection (e.g. either ≥ 1 misrouted projection or misrouting plus loss affecting ≥2 projections) appear specific to DNT1, and misroutings and/or loss of ≥1 projection are specific for DNT2 and Spz function. There are some affected axons also in wild-type and in controls, which we take to be normal (threshold line in Figure 7). Thus we asked whether the incidence of the misrouting and multiple-projections-affected phenotypes increases in experimental genotypes compared to wild-type or to controls, by applying a Chi-square test (planned comparisons). CI 95%, p values are: There is a significant increase in the incidence of ISNb/d and SNa misrouting phenotypes in DNT1^41^ mutants (DNT1^41^ vs wt:  χ^2^ p=0.000; SNa χ^2^ p=0.015. DNT1^41^ /DNT1^55^  vs wt:  χ^2^ p=0.000; Sna: χ^2^ p=0.002. DNT1^41^/ Df(3L)ED4342 vs wt ISNb/d χ^2^ p=0.000, SNa χ^2^ p=0.000) and upon RNAi targeted to the muscle (24BGAL4/UASpWCKRNAi;Df(3L)ED4342 vs wt ISNb/d χ^2^ p=0.001; 24BGAL4/UASCKRNAi;Df(3L)ED4342 vs wt SNa χ^2^ p=0.000; 24BGAL4/UASproRNAi;Df(3L)ED4342 vs wt ISNb/d χ^2^ p=0.000, SNa χ^2^ p=0.000). There is no significant difference between Df(3L)ED4342/+ control embryos and wild-type (ISNb/d χ^2^ p=0.367). There is a significant difference between DNT1^41^/ Df(3L)ED4342 and Df(3L)ED4342/+ control embryos (ISNb/d χ^2^ p=0.000, SNa χ^2^ p=0.035). There is no significant difference between controls expressing DNT1-RNAi in all neurons and wild-type (elavGAL4/UASpWCKRNAi vs wt ISNb/d χ^2^ p=0.386; SNa  χ^2^ p=0.376; elavGAL4/UASproRNAi vs wt SNa  χ^2^ p=0.575). There is a significant increase in the incidence of ISNb/d and SNa misrouting phenotypes when comparing targeted RNAi to the muscle with RNAi expressed in all neurons (24BGAL4/UASpWCKRNAi;Df(3L)ED4342 vs elavGAL4/UASpWCKRNAi ISNb/d χ^2^ p=0.002; 24BGAL4/ UASCKRNAi;Df(3L)ED4342 vs elavGAL4/UASCKRNAi SNa χ^2^ p=0.000; 24BGAL4/UASproRNAi;Df(3L)ED4342 vs elavGAL4/UASproRNAi ISNb/d χ^2^ p=0.03, SNa χ^2^ p=0.000). There is a significant increase in the incidence of misrouting phenotypes upon ectopic expression of DNT1 in the muscle (24BGAl4/UASCysknot3’tail vs wt: ISNb/d: χ^2^ p=0.000 SNa: χ^2^ p=0.000). Figure 9C: There is a significant increase in the incidence of misrouting phenotypes in *spz^2^* vs wt (Sna χ^2^  p=0.000; DNT1^41^/ Df(3L)ED4342 vs *spz^2^DNT1^41^* double mutants: Sna χ^2^ p=0.000). There is no significant difference in SNa misrouting phenotypes in *spz^2^* vs *spz^2^DNT1^41^* (χ^2^ p=0.344).

Figure 9D: There is a significant increase in the incidence of ISNb/d misrouting and multiple projection phenotypes in DNT2 mutant embryos compared to wild-type (*DNT2^e0344^/Dfr6092* vs *wt* χ^2^  p=0.05). There is no significant difference between *DNT1* and *DNT1 DNT2* double mutants (*DNT1^41^* vs *DNT1^41^DNT2^e0344^/ DNT1^41^Dfr6092* Fisher’s Exact test p=0.06). There is a significant increase in ISNb/d phenotypes in the triple mutants *DNT1^41^ DNT2^e0344^spz^2^* compared to *DNT1^41^* χ^2^  p=0.000) and compared to the double mutants *DNT1^41^ DNT2^e0344^* (*DNT1^41^ DNT2^e0344^spz^2^* vs *DNT1^41^DNT2^e0344^/ DNT1^41^Dfr6092* χ^2^  p=0.02).

***Films of adult locomotion phenotypes***

# Adult flies were sent to sleep in CO_2_ and their wings were removed to prevent flight. After 30 min, they were placed in the centre of a small Petri-dish. Normal flies run to the edge, climb it and walk fast on the rim. The rim is thin thus problems with locomotion or propioception are more apparent. Flies were filmed using a Motic cam 2000 2.0M pixel USB 2.0 camera mounted on a Leica MZ8 microscope using the Motic Images Plus 2.0 software with a Dell PC. Films were compressed and converted Windows Media Video (wmv) files using Windows Movie Maker and then converted to mpeg1 (mpg) files using Any Video Converter Free software.

# SUPPLEMENTARY MATERIAL AND METHODS REFERENCES

# 1. Mizuguchi, K., et al., *Getting knotted: a model for the structure and activation of Spätzle.* TIBS, 1998. 23: p. 239-361.

# 2. Shi, J., T.L. Blundell, and K. Mizuguchi, *FUGUE:Sequence-structure homology recognition using environment-specific substitution tables and structure-dependent gap penalties.* J.Mol.Biol., 2001. 310: p. 243-257.

# 3. Mizuguchi, K., et al., *HOMSTRAD: a database of protein structure alignments for homologous families.* Protein Sci, 1998. 7: p. 2469-2471.

# 4. Hallböök, F., *Lampetra fluvialitis neurotrophin homolog, descendant of a neurotorphin ancestor, discloses the early molecular evolution of neurotrophins in the vertebrate subphylum.* Journal of Neuroscience, 1998. 18: p. 8700-8711.

# 5. Hallböök, F., et al., *Formation and evolution of the chordate neurotorphin and Trk receptor genes.* Brain, Behaviour and Evolution, 2006. 68: p. 133-144.

# 6. Robinson, R.C., et al., *Structure of the brain-derived neurotorphic factor/neurotrophin 3 heterodimer.* Biochemistry, 1995. 34: p. 4139-4146.

# 7. McDonald, N.Q., et al., *New protein fold revealed by a 2.3-A resolution crystal structure of nerve growth factor.* Nature, 1991. 354: p. 411-414.

# 8. Robinson, R.C., et al., *The structures of the neurotrophin 4 homodimer and the brain-derived neurotrophic factor/neurotrophin 4 heterodimer reveal a common Trk-binding site.* Protein Sci., 1999. 8: p. 2589-2597.

# 9. Gouet, P., et al., *ESPript: multiple sequence alignments in PostScript.* Bioinformatics, 1999. 15: p. 305-308.

# 10. Sali, A. and T.L. Blundell, *Comparative protein modelling by satisfaction of spatial restraints.* J Mol Biol, 1993. 234: p. 779-815.

# 11. Jones, D.T., W.R. Taylor, and J.M. Thornton, *The rapid generationof mutation data matrices from protein sequences.* CABIOS, 1992. 8: p. 275-282.

# 12. Weber, N.R., et al., *Role of the Spätzle pro-domain in the generation of an active Toll receptor ligand.* Journal of Biological Chemistry, 2007. 282: p. 13522-13531.

# 13. Chen, Z.-Y., et al., *Genetic variant BDNF (Val66Met) polymorphism alters anxiety-related behaviour.* Science, 2006. 314: p. 140-143.

# 14. Bischof, J., et al., *An optimised transgenesis system for Drosophila using germ-line-specific phi31 integrases.* PNAS, 2007. 104: p. 3312-3317.

# 15. Sweeney, S.T., et al., *Functional cell ablation*, in *Drosophila Protocosl*, Sullivan, W., M. Ashburner, and R.S. Hawley, Editors. 2000, Cold Spring Harbour Laboratory Press. p. 449-477.

# 16. White, K., et al., *Genetic control of programmed cell death in Drosophila.* Science, 1994. 264: p. 677-683.
